# Supplementary material for: Large-scale network integration in the human brain tracks temporal fluctuations in memory encoding performance
Source: eLife. 2018 Jun 18;7:e32696. doi: 10.7554/eLife.32696 (PMC6039182; doi:10.7554/eLife.32696)
Supplement: Supplementary file 1. [file elife-32696-supp1.docx]

**Supplementary file 1**

**Supplementary file 1A. Details of 21 ROIs related with memory encoding.**

| **ROI ID** | **Name of ROI** | **Hemisphere** | **MNI coordinates** | | |
| --- | --- | --- | --- | --- | --- |
|  |  |  | ***x*** | ***y*** | ***z*** |
| Subsequent memory effect (SME) related areas | | | | | |
| 1 | Inferior frontal cortex, premotor cortex, precentral gyrus | L | -46 | 26 | 19 |
| 2 | Inferior frontal cortex, premotor cortex | R | 48 | 7 | 33 |
| 3 | Pre-supplementary motor cortex | L | -6 | 14 | 53 |
| 4 | Hippocampus, parahippocampal gyrus, amygdala | L | -22 | -9 | -20 |
| 5 | Hippocampus, parahippocampal gyrus, amygdala | R | 18 | -7 | -19 |
| 6 | Fusiform gyrus | L | -42 | -46 | -29 |
| 7 | Fusiform gyrus | R | 44 | -53 | -20 |
| 8 | Intraparietal sulcus | L | -28 | -80 | 35 |
| 9 | Intraparietal sulcus | R | 26 | -66 | 47 |
| 10 | Middle occipital gyrus | R | 28 | -89 | 2 |
| 11 | Inferior occipital gyrus | L | -32 | -92 | -12 |
| Subsequent forgotten effect (SFE) related areas | | | | | |
| 12 | Frontal pole | L | -26 | 54 | 27 |
| 13 | Frontal pole | R | 38 | 46 | 22 |
| 14 | Superior frontal cortex | L | -36 | 29 | 43 |
| 15 | Superior frontal cortex | R | 24 | 35 | 41 |
| 16 | Superior frontal cortex | R | 34 | 31 | 47 |
| 17 | Anterior cingulate cortex, ventromedial prefrontal cortex | - | 0 | 45 | 5 |
| 18 | Superior temporal gyrus | L | -48 | -14 | -10 |
| 19 | Posterior cingulate cortex, precuneus | - | 0 | -29 | 40 |
| 20 | Temporoparietal junction | L | -53 | -59 | 32 |
| 21 | Temporoparietal junction | R | 55 | -49 | 30 |

*Notes*. ROIs are derived from (Kim 2011).Table 2 and 6. Coordinates are converted from Talairach to MNI space.

**Supplementary file 1B. Differences in trial-related activation between HH and Miss trials in 21 ROIs related with memory encoding.**

| **SME** | | | | **SFE** | | | |
| --- | --- | --- | --- | --- | --- | --- | --- |
| **Name of ROI** | **H** | ***z*** | ***P*** | **Name of ROI** | **H** | ***z*** | ***P*** |
| Inferior frontal cortex (IFC),  Premotor cortex (PMC),  precentral gyrus | L | 4.1033 | 0.00004 | Frontal pole (FP) | L | -1.3857 | 0.16584 |
| Inferior frontal cortex,  Premotor cortex | R | 3.8611 | 0.00011 | FP | R | -3.2692 | 0.00108 |
| Pre-supplementary motor cortex | L | 3.4306 | 0.00060 | Superior frontal cortex | L | -2.0853 | 0.03704 |
| Hippocampus (HCP),  Parahippocampal gyrus (PHG),  amygdala | L | 3.9688 | 0.00007 | Superior frontal cortex | R | -3.4844 | 0.00049 |
| HCP, PHG, Amygdala | R | 4.3455 | 0.00001 | Superior frontal cortex | R | -3.4575 | 0.00055 |
| Fusiform gyrus | L | 3.9419 | 0.00008 | Anterior cingulate cortex (ACC),  ventromedial prefrontal cortex (vmPFC) | - | -4.3724 | 0.00001 |
| Fusiform gyrus | R | 4.3724 | 0.00001 | Superior temporal gyrus | L | -2.0584 | 0.03955 |
| Intraparietal sulcus (IPS) | L | 3.7535 | 0.00017 | Posterior cingulate cortex (PCC) ,  precuneus | - | -3.6997 | 0.00022 |
| IPS | R | 4.0495 | 0.00005 | Temporoparietal junction (TPJ) | L | -3.1347 | 0.00172 |
| Middle occipital gyrus (MOG) | R | 4.2917 | 0.00002 | TPJ | R | -4.3186 | 0.00002 |
| Inferior occipital gyrus | L | 3.8611 | 0.00011 |  |  |  |  |

**Supplementary file 1C.** **224 ROIs included in 10 subnetworks of the Power atlas.**

| **ROI ID** | **Name of ROI**  **(Automated Anatomical Label)** | **Hemisphere** | **MNI coordinates** | | | **Assigned network** |
| --- | --- | --- | --- | --- | --- | --- |
|  |  |  | ***x*** | ***y*** | ***z*** |  |
| 1 | Precuneus_L | L | -7 | -52 | 61 | SMN |
| 2 | undefined | L | -14 | -18 | 40 | SMN |
| 3 | Supp_Motor_Area_L | R | 0 | -15 | 47 | SMN |
| 4 | Supp_Motor_Area_R | R | 10 | -2 | 45 | SMN |
| 5 | Paracentral_Lobule_L | L | -7 | -21 | 65 | SMN |
| 6 | Paracentral_Lobule_L | L | -7 | -33 | 72 | SMN |
| 7 | Postcentral_R | R | 13 | -33 | 75 | SMN |
| 8 | Parietal_Inf_L | L | -54 | -23 | 43 | SMN |
| 9 | Precentral_R | R | 29 | -17 | 71 | SMN |
| 10 | Precuneus_R | R | 10 | -46 | 73 | SMN |
| 11 | Postcentral_L | L | -23 | -30 | 72 | SMN |
| 12 | Precentral_L | L | -40 | -19 | 54 | SMN |
| 13 | Postcentral_R | R | 29 | -39 | 59 | SMN |
| 14 | Postcentral_R | R | 50 | -20 | 42 | SMN |
| 15 | Postcentral_L | L | -38 | -27 | 69 | SMN |
| 16 | undefined | R | 20 | -29 | 60 | SMN |
| 17 | Precentral_R | R | 44 | -8 | 57 | SMN |
| 18 | Postcentral_L | L | -29 | -43 | 61 | SMN |
| 19 | Supp_Motor_Area_R | R | 10 | -17 | 74 | SMN |
| 20 | Postcentral_R | R | 22 | -42 | 69 | SMN |
| 21 | Postcentral_L | L | -45 | -32 | 47 | SMN |
| 22 | Postcentral_L | L | -21 | -31 | 61 | SMN |
| 23 | Paracentral_Lobule_L | L | -13 | -17 | 75 | SMN |
| 24 | Precentral_R | R | 42 | -20 | 55 | SMN |
| 25 | undefined | L | -38 | -15 | 69 | SMN |
| 26 | Parietal_Sup_L | L | -16 | -46 | 73 | SMN |
| 27 | Paracentral_Lobule_R | R | 2 | -28 | 60 | SMN |
| 28 | Supp_Motor_Area_R | R | 3 | -17 | 58 | SMN |
| 29 | Precentral_R | R | 38 | -17 | 45 | SMN |
| 30 | Postcentral_R | R | 47 | -30 | 49 | SMN |
| 31 | Postcentral_L | L | -49 | -11 | 35 | SMN |
| 32 | Insula_R | R | 36 | -9 | 14 | SMN |
| 33 | Postcentral_R | R | 51 | -6 | 32 | SMN |
| 34 | Postcentral_L | L | -53 | -10 | 24 | SMN |
| 35 | Postcentral_R | R | 66 | -8 | 25 | SMN |
| 36 | Supp_Motor_Area_L | L | -3 | 2 | 53 | CON |
| 37 | SupraMarginal_R | R | 54 | -28 | 34 | CON |
| 38 | Frontal_Sup_R | R | 19 | -8 | 64 | CON |
| 39 | Frontal_Sup_L | L | -16 | -5 | 71 | CON |
| 40 | Cingulum_Mid_L | L | -10 | -2 | 42 | CON |
| 41 | undefined | R | 37 | 1 | -4 | CON |
| 42 | Supp_Motor_Area_R | R | 13 | -1 | 70 | CON |
| 43 | Supp_Motor_Area_R | R | 7 | 8 | 51 | CON |
| 44 | Rolandic_Oper_L | L | -45 | 0 | 9 | CON |
| 45 | Insula_R | R | 49 | 8 | -1 | CON |
| 46 | undefined | L | -34 | 3 | 4 | CON |
| 47 | Temporal_Pole_Sup_L | L | -51 | 8 | -2 | CON |
| 48 | Cingulum_Mid_L | L | -5 | 18 | 34 | CON |
| 49 | undefined | R | 36 | 10 | 1 | CON |
| 50 | undefined | R | 32 | -26 | 13 | AUD |
| 51 | Temporal_Sup_R | R | 65 | -33 | 20 | AUD |
| 52 | Temporal_Sup_R | R | 58 | -16 | 7 | AUD |
| 53 | Rolandic_Oper_L | L | -38 | -33 | 17 | AUD |
| 54 | Temporal_Sup_L | L | -60 | -25 | 14 | AUD |
| 55 | Temporal_Sup_L | L | -49 | -26 | 5 | AUD |
| 56 | Rolandic_Oper_R | R | 43 | -23 | 20 | AUD |
| 57 | SupraMarginal_L | L | -50 | -34 | 26 | AUD |
| 58 | SupraMarginal_L | L | -53 | -22 | 23 | AUD |
| 59 | Rolandic_Oper_L | L | -55 | -9 | 12 | AUD |
| 60 | Rolandic_Oper_R | R | 56 | -5 | 13 | AUD |
| 61 | Postcentral_R | R | 59 | -17 | 29 | AUD |
| 62 | undefined | L | -30 | -27 | 12 | AUD |
| 63 | Occipital_Mid_L | L | -41 | -75 | 26 | DMN |
| 64 | Rectus_R | R | 8 | 48 | -15 | DMN |
| 65 | Precuneus_L | L | -13 | -40 | 1 | DMN |
| 66 | Temporal_Mid_L | L | -46 | -61 | 21 | DMN |
| 67 | Occipital_Mid_R | R | 43 | -72 | 28 | DMN |
| 68 | Temporal_Pole_Mid_L | L | -44 | 12 | -34 | DMN |
| 69 | Temporal_Pole_Mid_R | R | 46 | 16 | -30 | DMN |
| 70 | Temporal_Mid_L | L | -68 | -23 | -16 | DMN |
| 71 | Angular_L | L | -44 | -65 | 35 | DMN |
| 72 | Angular_L | L | -39 | -75 | 44 | DMN |
| 73 | Precuneus_L | L | -7 | -55 | 27 | DMN |
| 74 | Precuneus_R | R | 6 | -59 | 35 | DMN |
| 75 | Precuneus_L | L | -11 | -56 | 16 | DMN |
| 76 | Precuneus_L | L | -3 | -49 | 13 | DMN |
| 77 | Cingulum_Mid_R | R | 8 | -48 | 31 | DMN |
| 78 | Precuneus_R | R | 15 | -63 | 26 | DMN |
| 79 | Cingulum_Mid_L | L | -2 | -37 | 44 | DMN |
| 80 | Precuneus_R | R | 11 | -54 | 17 | DMN |
| 81 | Angular_R | R | 52 | -59 | 36 | DMN |
| 82 | Frontal_Sup_R | R | 23 | 33 | 48 | DMN |
| 83 | Frontal_Sup_Medial_L | L | -10 | 39 | 52 | DMN |
| 84 | Frontal_Sup_L | L | -16 | 29 | 53 | DMN |
| 85 | Frontal_Mid_L | L | -35 | 20 | 51 | DMN |
| 86 | Frontal_Sup_R | R | 22 | 39 | 39 | DMN |
| 87 | Frontal_Sup_R | R | 13 | 55 | 38 | DMN |
| 88 | Frontal_Sup_L | L | -10 | 55 | 39 | DMN |
| 89 | Frontal_Sup_L | L | -20 | 45 | 39 | DMN |
| 90 | Frontal_Sup_Medial_R | R | 6 | 54 | 16 | DMN |
| 91 | Frontal_Sup_Medial_R | R | 6 | 64 | 22 | DMN |
| 92 | Cingulum_Ant_L | L | -7 | 51 | -1 | DMN |
| 93 | Frontal_Sup_Medial_R | R | 9 | 54 | 3 | DMN |
| 94 | Frontal_Med_Orb_L | L | -3 | 44 | -9 | DMN |
| 95 | Frontal_Med_Orb_R | R | 8 | 42 | -5 | DMN |
| 96 | Cingulum_Ant_L | L | -11 | 45 | 8 | DMN |
| 97 | Frontal_Sup_Medial_L | L | -2 | 38 | 36 | DMN |
| 98 | Cingulum_Ant_L | L | -3 | 42 | 16 | DMN |
| 99 | Frontal_Sup_L | L | -20 | 64 | 19 | DMN |
| 100 | Frontal_Sup_Medial_L | L | -8 | 48 | 23 | DMN |
| 101 | Temporal_Mid_R | R | 65 | -12 | -19 | DMN |
| 102 | Temporal_Mid_L | L | -56 | -13 | -10 | DMN |
| 103 | Temporal_Mid_L | L | -58 | -30 | -4 | DMN |
| 104 | Temporal_Mid_R | R | 65 | -31 | -9 | DMN |
| 105 | Temporal_Mid_L | L | -68 | -41 | -5 | DMN |
| 106 | Frontal_Sup_Medial_R | R | 13 | 30 | 59 | DMN |
| 107 | Cingulum_Ant_R | R | 12 | 36 | 20 | DMN |
| 108 | Temporal_Mid_R | R | 52 | -2 | -16 | DMN |
| 109 | ParaHippocampal_L | L | -26 | -40 | -8 | DMN |
| 110 | Fusiform_R | R | 27 | -37 | -13 | DMN |
| 111 | Fusiform_L | L | -34 | -38 | -16 | DMN |
| 112 | Cerebelum_Crus1_R | R | 28 | -77 | -32 | DMN |
| 113 | Temporal_Pole_Mid_R | R | 52 | 7 | -30 | DMN |
| 114 | Temporal_Mid_L | L | -53 | 3 | -27 | DMN |
| 115 | Angular_R | R | 47 | -50 | 29 | DMN |
| 116 | Temporal_Mid_L | L | -49 | -42 | 1 | DMN |
| 117 | Frontal_Inf_Orb_L | L | -46 | 31 | -13 | DMN |
| 118 | Frontal_Inf_Orb_R | R | 49 | 35 | -12 | DMN |
| 119 | Lingual_R | R | 18 | -47 | -10 | VIN |
| 120 | Occipital_Mid_R | R | 40 | -72 | 14 | VIN |
| 121 | Calcarine_R | R | 8 | -72 | 11 | VIN |
| 122 | Calcarine_L | L | -8 | -81 | 7 | VIN |
| 123 | Occipital_Mid_L | L | -28 | -79 | 19 | VIN |
| 124 | Lingual_R | R | 20 | -66 | 2 | VIN |
| 125 | Occipital_Mid_L | L | -24 | -91 | 19 | VIN |
| 126 | Fusiform_R | R | 27 | -59 | -9 | VIN |
| 127 | Lingual_L | L | -15 | -72 | -8 | VIN |
| 128 | Calcarine_L | L | -18 | -68 | 5 | VIN |
| 129 | Occipital_Inf_R | R | 43 | -78 | -12 | VIN |
| 130 | Occipital_Inf_L | L | -47 | -76 | -10 | VIN |
| 131 | Occipital_Sup_L | L | -14 | -91 | 31 | VIN |
| 132 | Cuneus_R | R | 15 | -87 | 37 | VIN |
| 133 | Occipital_Mid_R | R | 29 | -77 | 25 | VIN |
| 134 | Lingual_R | R | 20 | -86 | -2 | VIN |
| 135 | Cuneus_R | R | 15 | -77 | 31 | VIN |
| 136 | Lingual_L | L | -16 | -52 | -1 | VIN |
| 137 | Temporal_Inf_R | R | 42 | -66 | -8 | VIN |
| 138 | Occipital_Sup_R | R | 24 | -87 | 24 | VIN |
| 139 | Cuneus_R | R | 6 | -72 | 24 | VIN |
| 140 | Occipital_Mid_L | L | -42 | -74 | 0 | VIN |
| 141 | Fusiform_R | R | 26 | -79 | -16 | VIN |
| 142 | Cuneus_L | L | -16 | -77 | 34 | VIN |
| 143 | Cuneus_L | L | -3 | -81 | 21 | VIN |
| 144 | Occipital_Mid_L | L | -40 | -88 | -6 | VIN |
| 145 | Occipital_Mid_R | R | 37 | -84 | 13 | VIN |
| 146 | Calcarine_R | R | 6 | -81 | 6 | VIN |
| 147 | Occipital_Mid_L | L | -26 | -90 | 3 | VIN |
| 148 | Fusiform_L | L | -33 | -79 | -13 | VIN |
| 149 | Occipital_Mid_R | R | 37 | -81 | 1 | VIN |
| 150 | Precentral_L | L | -44 | 2 | 46 | FPN |
| 151 | Frontal_Inf_Tri_R | R | 48 | 25 | 27 | FPN |
| 152 | Frontal_Inf_Tri_L | L | -47 | 11 | 23 | FPN |
| 153 | Parietal_Inf_L | L | -53 | -49 | 43 | FPN |
| 154 | Frontal_Mid_L | L | -23 | 11 | 64 | FPN |
| 155 | Temporal_Inf_R | R | 58 | -53 | -14 | FPN |
| 156 | Frontal_Sup_Orb_R | R | 24 | 45 | -15 | FPN |
| 157 | Frontal_Mid_Orb_R | R | 34 | 54 | -13 | FPN |
| 158 | Precentral_R | R | 47 | 10 | 33 | FPN |
| 159 | Precentral_L | L | -41 | 6 | 33 | FPN |
| 160 | Frontal_Mid_R | R | 38 | 43 | 15 | FPN |
| 161 | Parietal_Inf_R | R | 49 | -42 | 45 | FPN |
| 162 | Parietal_Inf_L | L | -28 | -58 | 48 | FPN |
| 163 | Parietal_Inf_R | R | 44 | -53 | 47 | FPN |
| 164 | Frontal_Mid_R | R | 32 | 14 | 56 | FPN |
| 165 | Angular_R | R | 37 | -65 | 40 | FPN |
| 166 | Parietal_Inf_L | L | -42 | -55 | 45 | FPN |
| 167 | Frontal_Mid_R | R | 40 | 18 | 40 | FPN |
| 168 | Frontal_Mid_L | L | -34 | 55 | 4 | FPN |
| 169 | Frontal_Mid_Orb_L | L | -42 | 45 | -2 | FPN |
| 170 | Angular_R | R | 33 | -53 | 44 | FPN |
| 171 | Frontal_Mid_Orb_R | R | 43 | 49 | -2 | FPN |
| 172 | Frontal_Inf_Tri_L | L | -42 | 25 | 30 | FPN |
| 173 | Frontal_Sup_Medial_L | L | -3 | 26 | 44 | FPN |
| 174 | Cingulum_Mid_R | R | 11 | -39 | 50 | SAN |
| 175 | Parietal_Inf_R | R | 55 | -45 | 37 | SAN |
| 176 | Precentral_R | R | 42 | 0 | 47 | SAN |
| 177 | Frontal_Mid_R | R | 31 | 33 | 26 | SAN |
| 178 | Frontal_Inf_Tri_R | R | 48 | 22 | 10 | SAN |
| 179 | Insula_L | L | -35 | 20 | 0 | SAN |
| 180 | Insula_R | R | 36 | 22 | 3 | SAN |
| 181 | Frontal_Inf_Orb_R | R | 37 | 32 | -2 | SAN |
| 182 | Insula_R | R | 34 | 16 | -8 | SAN |
| 183 | undefined | L | -11 | 26 | 25 | SAN |
| 184 | Supp_Motor_Area_L | L | -1 | 15 | 44 | SAN |
| 185 | Frontal_Mid_L | L | -28 | 52 | 21 | SAN |
| 186 | Cingulum_Ant_L | L | 0 | 30 | 27 | SAN |
| 187 | Cingulum_Mid_R | R | 5 | 23 | 37 | SAN |
| 188 | Cingulum_Ant_R | R | 10 | 22 | 27 | SAN |
| 189 | Frontal_Mid_R | R | 31 | 56 | 14 | SAN |
| 190 | Frontal_Mid_R | R | 26 | 50 | 27 | SAN |
| 191 | Frontal_Mid_L | L | -39 | 51 | 17 | SAN |
| 192 | Thalamus_R | R | 6 | -24 | 0 | SUB |
| 193 | Thalamus_L | L | -2 | -13 | 12 | SUB |
| 194 | Thalamus_L | L | -10 | -18 | 7 | SUB |
| 195 | Thalamus_R | R | 12 | -17 | 8 | SUB |
| 196 | undefined | L | -5 | -28 | -4 | SUB |
| 197 | Putamen_L | L | -22 | 7 | -5 | SUB |
| 198 | undefined | L | -15 | 4 | 8 | SUB |
| 199 | Putamen_R | R | 31 | -14 | 2 | SUB |
| 200 | Putamen_R | R | 23 | 10 | 1 | SUB |
| 201 | Putamen_R | R | 29 | 1 | 4 | SUB |
| 202 | undefined | L | -31 | -11 | 0 | SUB |
| 203 | undefined | R | 15 | 5 | 7 | SUB |
| 204 | undefined | R | 9 | -4 | 6 | SUB |
| 205 | Supp_Motor_Area_L | L | -10 | 11 | 67 | VAN |
| 206 | Temporal_Sup_R | R | 54 | -43 | 22 | VAN |
| 207 | Temporal_Mid_L | L | -56 | -50 | 10 | VAN |
| 208 | Temporal_Sup_L | L | -55 | -40 | 14 | VAN |
| 209 | Temporal_Sup_R | R | 52 | -33 | 8 | VAN |
| 210 | Temporal_Mid_R | R | 51 | -29 | -4 | VAN |
| 211 | Temporal_Sup_R | R | 56 | -46 | 11 | VAN |
| 212 | Frontal_Inf_Tri_R | R | 53 | 33 | 1 | VAN |
| 213 | Frontal_Inf_Tri_L | L | -49 | 25 | -1 | VAN |
| 214 | Precuneus_R | R | 10 | -62 | 61 | DAN |
| 215 | Temporal_Mid_L | L | -52 | -63 | 5 | DAN |
| 216 | Parietal_Sup_R | R | 22 | -65 | 48 | DAN |
| 217 | Temporal_Mid_R | R | 46 | -59 | 4 | DAN |
| 218 | Parietal_Sup_R | R | 25 | -58 | 60 | DAN |
| 219 | Parietal_Inf_L | L | -33 | -46 | 47 | DAN |
| 220 | Occipital_Mid_L | L | -27 | -71 | 37 | DAN |
| 221 | Precentral_L | L | -32 | -1 | 54 | DAN |
| 222 | Temporal_Inf_L | L | -42 | -60 | -9 | DAN |
| 223 | Parietal_Sup_L | L | -17 | -59 | 64 | DAN |
| 224 | Precentral_R | R | 29 | -5 | 54 | DAN |

*Note*. SMN, sensorimotor networks; CON, cingulo-opercular network; AUD, auditory network; DMN, default mode network; VIN, visual network; FPN, fronto-parietal network; SAN, salience network; SUB, subcortical nodes; VAN, ventral attention network; DAN, dorsal attention network.

**Supplementary file 1D. 98 connections showing significant increase in FC during the high encoding state.**

| **Subnetwork pairs** | | **ROI pairs** | | ***z*** | ***P*** |
| --- | --- | --- | --- | --- | --- |
| DMN | SMN | Frontal_Sup_Medial_R | Postcentral_L | 3.5114 | 0.0004 |
|  | DMN | Frontal_Sup_L | Angular_L | 3.4844 | 0.0005 |
|  |  | Frontal_Sup_R | Temporal_Pole_Mid_L | 3.2423 | 0.0012 |
|  |  | Frontal_Sup_Medial_R | Frontal_Sup_Medial_L | 3.2961 | 0.0010 |
|  |  | Temporal_Mid_L | Frontal_Sup_R | 3.2154 | 0.0013 |
|  |  | Cingulum_Ant_R | Frontal_Sup_Medial_L | 3.5921 | 0.0003 |
|  |  | Temporal_Mid_L | Frontal_Sup_Medial_R | 3.1885 | 0.0014 |
| VIN | SMN | Occipital_Inf_R | undefined | 3.2423 | 0.0012 |
|  |  | Occipital_Inf_R | Insula_R | 3.2961 | 0.0010 |
|  |  | Fusiform_R | Parietal_Inf_L | 3.3768 | 0.0007 |
|  |  | Fusiform_R | Postcentral_L | 3.9957 | 0.0001 |
|  |  | Fusiform_R | Precentral_R | 3.5114 | 0.0004 |
|  |  | Fusiform_R | Postcentral_R | 3.1616 | 0.0016 |
|  |  | Fusiform_R | Postcentral_L | 3.8073 | 0.0001 |
|  |  | Fusiform_R | Insula_R | 3.9150 | 0.0001 |
|  |  | Fusiform_R | Postcentral_L | 3.6459 | 0.0003 |
|  |  | Occipital_Mid_L | Postcentral_R | 4.0226 | 0.0001 |
|  |  | Occipital_Mid_L | Precentral_R | 3.2692 | 0.0011 |
|  |  | Occipital_Mid_L | Postcentral_R | 3.8880 | 0.0001 |
|  |  | Occipital_Mid_L | Precentral_R | 3.4844 | 0.0005 |
|  |  | Occipital_Mid_L | Postcentral_L | 3.5383 | 0.0004 |
|  |  | Occipital_Mid_L | Postcentral_L | 3.1616 | 0.0016 |
|  |  | Occipital_Mid_L | Paracentral_Lobule_L | 3.3499 | 0.0008 |
|  |  | Occipital_Mid_L | Precentral_R | 3.5114 | 0.0004 |
|  |  | Occipital_Mid_L | Precentral_R | 3.2154 | 0.0013 |
|  |  | Occipital_Mid_L | Parietal_Inf_L | 3.1885 | 0.0014 |
|  |  | Occipital_Mid_L | Postcentral_R | 3.2961 | 0.0010 |
|  |  | Occipital_Mid_L | Precentral_R | 3.4844 | 0.0005 |
|  |  | Occipital_Mid_L | Precentral_R | 3.3768 | 0.0007 |
|  |  | Fusiform_L | Postcentral_L | 3.8342 | 0.0001 |
|  |  | Fusiform_L | Precentral_R | 3.3230 | 0.0009 |
|  | VIN | Fusiform_R | Lingual_R | 3.6728 | 0.0002 |
|  |  | Fusiform_R | Fusiform_R | 3.5921 | 0.0003 |
|  |  | Fusiform_R | Occipital_Mid_R | 3.1616 | 0.0016 |
|  |  | Fusiform_R | Occipital_Sup_R | 3.1616 | 0.0016 |
|  |  | Fusiform_R | Cuneus_R | 3.4037 | 0.0007 |
|  |  | Cuneus_L | Fusiform_R | 3.6728 | 0.0002 |
|  |  | Occipital_Mid_R | Occipital_Mid_L | 3.6997 | 0.0002 |
|  | DMN | Occipital_Mid_L | Precuneus_L | 3.4575 | 0.0005 |
|  |  | Occipital_Mid_L | Fusiform_R | 3.2692 | 0.0011 |
|  | AUD | Fusiform_R | Temporal_Sup_R | 3.4306 | 0.0006 |
|  |  | Occipital_Mid_L | SupraMarginal_L | 3.2961 | 0.0010 |
|  |  | Occipital_Mid_L | Rolandic_Oper_L | 3.2423 | 0.0012 |
|  | CON | Lingual_R | Supp_Motor_Area_R | 3.2423 | 0.0012 |
|  |  | Occipital_Mid_L | Frontal_Sup_L | 3.4844 | 0.0005 |
| FPN | SMN | Parietal_Inf_L | Postcentral_R | 3.3230 | 0.0009 |
|  |  | Precentral_L | Postcentral_R | 3.1885 | 0.0014 |
|  |  | Frontal_Mid_L | Parietal_Sup_L | 3.6997 | 0.0002 |
|  | VIN | Frontal_Mid_R | Occipital_Sup_L | 3.4844 | 0.0005 |
| SAN | SMN | Frontal_Mid_L | Paracentral_Lobule_L | 3.5114 | 0.0004 |
|  |  | Frontal_Mid_L | Postcentral_R | 3.1885 | 0.0014 |
|  |  | Frontal_Mid_L | Precentral_L | 3.2961 | 0.0010 |
|  |  | Frontal_Mid_L | Postcentral_R | 3.1616 | 0.0016 |
|  |  | Frontal_Mid_L | Postcentral_R | 3.7804 | 0.0002 |
|  |  | Frontal_Mid_L | Paracentral_Lobule_R | 3.5114 | 0.0004 |
|  |  | Cingulum_Mid_R | Precentral_R | 3.5114 | 0.0004 |
|  |  | Frontal_Mid_L | Postcentral_R | 3.1616 | 0.0016 |
|  |  | Frontal_Mid_L | Postcentral_L | 3.3230 | 0.0009 |
|  |  | Frontal_Mid_L | undefined | 3.5383 | 0.0004 |
|  |  | Frontal_Mid_L | Postcentral_L | 3.3499 | 0.0008 |
|  |  | Frontal_Mid_L | Insula_R | 3.5114 | 0.0004 |
|  | FPN | Parietal_Inf_R | Frontal_Mid_Orb_L | 3.1616 | 0.0016 |
|  | VIN | Frontal_Mid_L | Cuneus_R | 3.2154 | 0.0013 |
|  | DMN | Parietal_Inf_R | Temporal_Pole_Mid_L | 3.1616 | 0.0016 |
|  |  | Parietal_Inf_R | Frontal_Sup_L | 3.3499 | 0.0008 |
|  |  | Parietal_Inf_R | Temporal_Pole_Mid_R | 3.5921 | 0.0003 |
|  |  | Parietal_Inf_R | Temporal_Mid_L | 3.2692 | 0.0011 |
|  |  | Frontal_Inf_Tri_R | Frontal_Sup_Medial_L | 3.5383 | 0.0004 |
|  |  | Frontal_Mid_L | Cingulum_Ant_L | 3.5921 | 0.0003 |
|  |  | Frontal_Mid_R | Temporal_Mid_L | 3.3499 | 0.0008 |
|  |  | Frontal_Mid_R | Cingulum_Mid_R | 3.1616 | 0.0016 |
|  | AUD | undefined | Temporal_Sup_R | 3.4306 | 0.0006 |
|  |  | Frontal_Mid_L | Rolandic_Oper_R | 3.3499 | 0.0008 |
|  |  | Cingulum_Ant_L | SupraMarginal_L | 3.3499 | 0.0008 |
|  | CON | Parietal_Inf_R | Cingulum_Mid_L | 3.5921 | 0.0003 |
| SUB | SMN | undefined | Paracentral_Lobule_R | 3.1616 | 0.0016 |
|  |  | undefined | Supp_Motor_Area_R | 3.2154 | 0.0013 |
|  |  | undefined | Paracentral_Lobule_L | 3.9688 | 0.0001 |
|  | SUB | Putamen_R | Thalamus_L | 3.1616 | 0.0016 |
|  | VIN | Thalamus_L | Cuneus_L | 3.2154 | 0.0013 |
|  |  | undefined | Fusiform_R | 3.2154 | 0.0013 |
|  | DMN | Thalamus_R | Frontal_Sup_Medial_L | 3.4037 | 0.0007 |
|  |  | Thalamus_R | Cerebelum_Crus1_R | 3.5383 | 0.0004 |
|  |  | Thalamus_L | Temporal_Pole_Mid_L | 3.2961 | 0.0010 |
|  |  | undefined | Frontal_Sup_R | 3.3768 | 0.0007 |
|  |  | undefined | Frontal_Sup_Medial_L | 3.2961 | 0.0010 |
|  |  | undefined | Temporal_Pole_Mid_R | 3.4575 | 0.0005 |
|  |  | undefined | Temporal_Mid_L | 3.3768 | 0.0007 |
|  | CON | undefined | Frontal_Sup_R | 3.1616 | 0.0016 |
| VAN | SUB | Temporal_Sup_L | undefined | 3.8880 | 0.0001 |
|  |  | Temporal_Sup_R | Thalamus_L | 3.1616 | 0.0016 |
|  | VIN | Temporal_Sup_L | Occipital_Mid_L | 3.2423 | 0.0012 |
|  | DMN | Temporal_Mid_L | Cerebelum_Crus1_R | 3.1616 | 0.0016 |
|  |  | Temporal_Mid_R | Cingulum_Ant_L | 3.3768 | 0.0007 |
|  |  | Frontal_Inf_Tri_L | Cingulum_Ant_L | 3.5921 | 0.0003 |
| DAN | SUB | Precuneus_R | undefined | 3.2961 | 0.0010 |
|  | FPN | Precentral_L | Angular_R | 3.1616 | 0.0016 |
|  | VIN | Precentral_R | Occipital_Mid_L | 3.1885 | 0.0014 |

**Supplementary file 1E. 687 connections showing significant decrease in FC during the high encoding state.**

| **Subnetwork pairs** | | **ROI pairs** | | ***z*** | ***P*** |
| --- | --- | --- | --- | --- | --- |
| SMN | SMN | Paracentral_Lobule_L | Paracentral_Lobule_L | -3.3230 | 0.0009 |
|  |  | Parietal_Inf_L | Supp_Motor_Area_L | -3.6459 | 0.0003 |
|  |  | Precentral_L | Precentral_R | -3.7266 | 0.0002 |
|  |  | Postcentral_L | Precentral_R | -3.6997 | 0.0002 |
|  |  | undefined | Parietal_Inf_L | -3.3768 | 0.0007 |
|  |  | undefined | Precentral_L | -3.3230 | 0.0009 |
|  |  | undefined | Postcentral_R | -3.1616 | 0.0016 |
|  |  | undefined | Postcentral_L | -3.4306 | 0.0006 |
|  |  | Precentral_R | Supp_Motor_Area_L | -3.2154 | 0.0013 |
|  |  | Postcentral_L | Precentral_R | -3.2692 | 0.0011 |
|  |  | Postcentral_L | undefined | -3.4575 | 0.0005 |
|  |  | Postcentral_R | Postcentral_L | -3.2423 | 0.0012 |
|  |  | Postcentral_L | Postcentral_R | -3.5114 | 0.0004 |
|  |  | Paracentral_Lobule_L | Postcentral_R | -3.7804 | 0.0002 |
|  |  | Precentral_R | Supp_Motor_Area_L | -3.1616 | 0.0016 |
|  |  | undefined | Precentral_R | -3.6997 | 0.0002 |
|  |  | Supp_Motor_Area_R | Precentral_L | -3.5652 | 0.0004 |
|  |  | Supp_Motor_Area_R | Postcentral_R | -3.2961 | 0.0010 |
|  |  | Supp_Motor_Area_R | Precentral_R | -3.5921 | 0.0003 |
|  |  | Supp_Motor_Area_R | Postcentral_L | -3.6459 | 0.0003 |
|  |  | Supp_Motor_Area_R | Postcentral_L | -3.2154 | 0.0013 |
|  |  | Precentral_R | Parietal_Inf_L | -3.1885 | 0.0014 |
|  |  | Precentral_R | Postcentral_L | -3.2154 | 0.0013 |
|  |  | Postcentral_L | Postcentral_L | -3.2692 | 0.0011 |
|  |  | Postcentral_L | Postcentral_L | -3.7266 | 0.0002 |
|  |  | Postcentral_L | Paracentral_Lobule_L | -3.4844 | 0.0005 |
|  |  | Postcentral_L | Supp_Motor_Area_R | -3.6728 | 0.0002 |
|  |  | Insula_R | Supp_Motor_Area_R | -3.2961 | 0.0010 |
|  |  | Insula_R | Postcentral_R | -3.2154 | 0.0013 |
|  |  | Insula_R | Precentral_R | -3.2961 | 0.0010 |
|  |  | Insula_R | Postcentral_L | -3.5652 | 0.0004 |
|  |  | Insula_R | Precentral_R | -3.1885 | 0.0014 |
|  |  | Postcentral_L | Supp_Motor_Area_R | -3.2692 | 0.0011 |
|  |  | Postcentral_R | Postcentral_R | -3.8342 | 0.0001 |
|  |  | Postcentral_R | Precentral_R | -3.4844 | 0.0005 |
| CON | SMN | SupraMarginal_R | Precentral_R | -3.2154 | 0.0013 |
|  |  | Frontal_Sup_R | Supp_Motor_Area_R | -3.1616 | 0.0016 |
|  |  | Frontal_Sup_L | Paracentral_Lobule_L | -3.7266 | 0.0002 |
|  |  | Cingulum_Mid_L | Insula_R | -3.8073 | 0.0001 |
|  |  | Supp_Motor_Area_R | Supp_Motor_Area_R | -3.2423 | 0.0012 |
|  |  | Rolandic_Oper_L | Paracentral_Lobule_L | -3.3499 | 0.0008 |
|  |  | Temporal_Pole_Sup_L | undefined | -3.4575 | 0.0005 |
|  | CON | Insula_R | undefined | -3.6459 | 0.0003 |
| AUD | SMN | Temporal_Sup_R | Supp_Motor_Area_R | -3.2423 | 0.0012 |
|  |  | Temporal_Sup_R | Precentral_R | -3.2961 | 0.0010 |
|  |  | Rolandic_Oper_L | Parietal_Inf_L | -3.4306 | 0.0006 |
|  |  | Rolandic_Oper_L | Supp_Motor_Area_R | -3.8880 | 0.0001 |
|  |  | Rolandic_Oper_L | Postcentral_R | -3.3499 | 0.0008 |
|  |  | Temporal_Sup_L | Precentral_R | -3.2692 | 0.0011 |
|  |  | Temporal_Sup_L | Postcentral_L | -3.6997 | 0.0002 |
|  |  | Temporal_Sup_L | Precentral_R | -3.4306 | 0.0006 |
|  |  | Temporal_Sup_L | Supp_Motor_Area_R | -3.3230 | 0.0009 |
|  |  | Temporal_Sup_L | Precentral_R | -4.0226 | 0.0001 |
|  |  | Temporal_Sup_L | undefined | -3.2961 | 0.0010 |
|  |  | Temporal_Sup_L | Postcentral_L | -3.3499 | 0.0008 |
|  |  | Temporal_Sup_L | Postcentral_L | -3.1616 | 0.0016 |
|  |  | SupraMarginal_L | Paracentral_Lobule_L | -3.2154 | 0.0013 |
|  |  | SupraMarginal_L | Precentral_R | -3.4037 | 0.0007 |
|  |  | SupraMarginal_L | Supp_Motor_Area_R | -3.1616 | 0.0016 |
|  |  | SupraMarginal_L | Paracentral_Lobule_L | -3.6190 | 0.0003 |
|  |  | SupraMarginal_L | Precentral_R | -3.3499 | 0.0008 |
|  |  | SupraMarginal_L | undefined | -3.1616 | 0.0016 |
|  |  | SupraMarginal_L | Supp_Motor_Area_R | -3.7266 | 0.0002 |
|  |  | SupraMarginal_L | Paracentral_Lobule_L | -3.8073 | 0.0001 |
|  |  | SupraMarginal_L | Precentral_R | -3.2692 | 0.0011 |
|  |  | Rolandic_Oper_L | Supp_Motor_Area_L | -3.2692 | 0.0011 |
|  |  | Rolandic_Oper_L | Supp_Motor_Area_R | -3.4306 | 0.0006 |
|  |  | Rolandic_Oper_L | Precentral_R | -3.4575 | 0.0005 |
|  |  | Rolandic_Oper_L | Postcentral_L | -3.4306 | 0.0006 |
|  |  | Rolandic_Oper_L | Supp_Motor_Area_R | -3.4575 | 0.0005 |
|  |  | Rolandic_Oper_R | Parietal_Inf_L | -3.3768 | 0.0007 |
|  |  | Rolandic_Oper_R | Postcentral_L | -3.6190 | 0.0003 |
|  |  | Rolandic_Oper_R | Paracentral_Lobule_L | -3.4037 | 0.0007 |
|  |  | Postcentral_R | Supp_Motor_Area_R | -3.2423 | 0.0012 |
|  |  | Postcentral_R | Precentral_R | -3.4037 | 0.0007 |
|  | AUD | Temporal_Sup_L | Temporal_Sup_R | -3.9150 | 0.0001 |
|  |  | SupraMarginal_L | Temporal_Sup_L | -3.4575 | 0.0005 |
|  |  | SupraMarginal_L | Temporal_Sup_L | -3.4844 | 0.0005 |
|  |  | Rolandic_Oper_R | Temporal_Sup_L | -3.6459 | 0.0003 |
|  |  | Postcentral_R | Rolandic_Oper_R | -3.2961 | 0.0010 |
|  | CON | Rolandic_Oper_L | Temporal_Pole_Sup_L | -3.5652 | 0.0004 |
| DMN | SMN | Occipital_Mid_L | Precuneus_L | -3.4844 | 0.0005 |
|  |  | Occipital_Mid_L | Supp_Motor_Area_L | -3.3499 | 0.0008 |
|  |  | Occipital_Mid_L | Supp_Motor_Area_R | -3.2692 | 0.0011 |
|  |  | Occipital_Mid_L | Parietal_Inf_L | -3.6459 | 0.0003 |
|  |  | Occipital_Mid_L | Postcentral_R | -3.1885 | 0.0014 |
|  |  | Occipital_Mid_L | Postcentral_L | -3.3768 | 0.0007 |
|  |  | Occipital_Mid_L | Postcentral_R | -3.3499 | 0.0008 |
|  |  | Rectus_R | Postcentral_R | -3.4037 | 0.0007 |
|  |  | Temporal_Mid_L | Precuneus_L | -3.5383 | 0.0004 |
|  |  | Temporal_Mid_L | Supp_Motor_Area_R | -3.6997 | 0.0002 |
|  |  | Temporal_Mid_L | Parietal_Inf_L | -3.8611 | 0.0001 |
|  |  | Temporal_Mid_L | Postcentral_R | -3.3230 | 0.0009 |
|  |  | Temporal_Mid_L | Postcentral_R | -4.0226 | 0.0001 |
|  |  | Temporal_Mid_L | Postcentral_L | -3.7804 | 0.0002 |
|  |  | Temporal_Mid_L | Supp_Motor_Area_R | -3.3768 | 0.0007 |
|  |  | Temporal_Mid_L | Postcentral_L | -3.4306 | 0.0006 |
|  |  | Temporal_Mid_L | Precentral_R | -3.8073 | 0.0001 |
|  |  | Temporal_Mid_L | Precentral_R | -3.4037 | 0.0007 |
|  |  | Temporal_Mid_L | Postcentral_R | -3.3768 | 0.0007 |
|  |  | Temporal_Mid_L | Postcentral_L | -3.1885 | 0.0014 |
|  |  | Occipital_Mid_R | Precuneus_L | -3.4306 | 0.0006 |
|  |  | Occipital_Mid_R | Supp_Motor_Area_R | -3.3499 | 0.0008 |
|  |  | Occipital_Mid_R | Postcentral_R | -3.2961 | 0.0010 |
|  |  | Occipital_Mid_R | Postcentral_R | -3.3768 | 0.0007 |
|  |  | Occipital_Mid_R | Postcentral_L | -3.1616 | 0.0016 |
|  |  | Temporal_Pole_Mid_R | Supp_Motor_Area_R | -3.2961 | 0.0010 |
|  |  | Temporal_Pole_Mid_R | Postcentral_R | -3.5114 | 0.0004 |
|  |  | Angular_L | Postcentral_R | -3.5114 | 0.0004 |
|  |  | Precuneus_L | Postcentral_R | -3.2961 | 0.0010 |
|  |  | Precuneus_L | Precuneus_L | -3.3230 | 0.0009 |
|  |  | Precuneus_L | Precentral_R | -3.3499 | 0.0008 |
|  |  | Precuneus_L | Postcentral_R | -3.5114 | 0.0004 |
|  |  | Precuneus_L | Postcentral_R | -3.4844 | 0.0005 |
|  |  | Precuneus_L | Postcentral_L | -3.2154 | 0.0013 |
|  |  | Precuneus_L | Precentral_R | -3.7266 | 0.0002 |
|  |  | Precuneus_L | Postcentral_R | -4.1033 | 0.0000 |
|  |  | Precuneus_L | Postcentral_L | -3.3499 | 0.0008 |
|  |  | Cingulum_Mid_R | Postcentral_R | -3.1885 | 0.0014 |
|  |  | Cingulum_Mid_R | Postcentral_R | -3.1616 | 0.0016 |
|  |  | Precuneus_R | Parietal_Inf_L | -3.1616 | 0.0016 |
|  |  | Precuneus_R | Postcentral_R | -3.7266 | 0.0002 |
|  |  | Precuneus_R | Postcentral_R | -3.9150 | 0.0001 |
|  |  | Precuneus_R | Insula_R | -3.4037 | 0.0007 |
|  |  | Frontal_Med_Orb_L | Postcentral_L | -3.7804 | 0.0002 |
|  |  | Frontal_Med_Orb_L | Postcentral_R | -3.3768 | 0.0007 |
|  |  | Temporal_Mid_R | Precuneus_L | -3.2154 | 0.0013 |
|  |  | Temporal_Mid_L | Supp_Motor_Area_R | -3.4575 | 0.0005 |
|  |  | Temporal_Mid_L | Postcentral_R | -3.5114 | 0.0004 |
|  |  | Temporal_Mid_L | Postcentral_L | -3.2423 | 0.0012 |
|  |  | Temporal_Mid_L | Insula_R | -3.1885 | 0.0014 |
|  |  | Temporal_Mid_L | Precuneus_L | -3.1616 | 0.0016 |
|  |  | Temporal_Mid_L | undefined | -3.2154 | 0.0013 |
|  |  | Temporal_Mid_L | Supp_Motor_Area_R | -3.7266 | 0.0002 |
|  |  | Temporal_Mid_L | Parietal_Inf_L | -3.1616 | 0.0016 |
|  |  | Temporal_Mid_L | Postcentral_R | -3.2961 | 0.0010 |
|  |  | Temporal_Mid_L | Postcentral_R | -3.2423 | 0.0012 |
|  |  | Temporal_Mid_L | Postcentral_L | -3.2154 | 0.0013 |
|  |  | Temporal_Mid_R | Supp_Motor_Area_R | -3.2423 | 0.0012 |
|  |  | Temporal_Mid_R | Postcentral_L | -3.5921 | 0.0003 |
|  |  | ParaHippocampal_L | Parietal_Inf_L | -3.1616 | 0.0016 |
|  |  | ParaHippocampal_L | Postcentral_R | -3.6997 | 0.0002 |
|  |  | ParaHippocampal_L | Postcentral_L | -3.4037 | 0.0007 |
|  |  | ParaHippocampal_L | Precentral_R | -3.4306 | 0.0006 |
|  |  | ParaHippocampal_L | Postcentral_R | -3.3768 | 0.0007 |
|  |  | Fusiform_R | Precuneus_L | -3.5114 | 0.0004 |
|  |  | Fusiform_R | Supp_Motor_Area_R | -3.5921 | 0.0003 |
|  |  | Fusiform_L | Postcentral_R | -3.6190 | 0.0003 |
|  |  | Fusiform_L | Postcentral_L | -3.5921 | 0.0003 |
|  |  | Temporal_Pole_Mid_R | Postcentral_R | -3.1885 | 0.0014 |
|  |  | Temporal_Pole_Mid_R | Paracentral_Lobule_L | -3.6997 | 0.0002 |
|  |  | Temporal_Mid_L | Postcentral_L | -3.2423 | 0.0012 |
|  |  | Temporal_Mid_L | Supp_Motor_Area_R | -3.9957 | 0.0001 |
|  |  | Temporal_Mid_L | Paracentral_Lobule_L | -3.2692 | 0.0011 |
|  |  | Temporal_Mid_L | Precentral_R | -3.1616 | 0.0016 |
|  |  | Temporal_Mid_L | Postcentral_L | -3.1885 | 0.0014 |
|  |  | Temporal_Mid_L | Supp_Motor_Area_R | -3.2692 | 0.0011 |
|  |  | Temporal_Mid_L | Precentral_R | -3.5114 | 0.0004 |
|  |  | Temporal_Mid_L | Insula_R | -3.1885 | 0.0014 |
|  |  | Frontal_Inf_Orb_L | Precuneus_R | -3.5383 | 0.0004 |
|  | DMN | Temporal_Pole_Mid_R | Temporal_Mid_L | -3.2154 | 0.0013 |
|  |  | Angular_R | Precuneus_L | -3.2154 | 0.0013 |
|  |  | Temporal_Mid_L | Precuneus_R | -3.5383 | 0.0004 |
|  |  | Temporal_Mid_R | Precuneus_L | -3.2961 | 0.0010 |
|  |  | Temporal_Mid_R | Frontal_Med_Orb_L | -3.2423 | 0.0012 |
|  |  | Temporal_Mid_R | Temporal_Mid_L | -3.2154 | 0.0013 |
|  |  | ParaHippocampal_L | Temporal_Mid_L | -3.3499 | 0.0008 |
|  |  | Temporal_Mid_L | Angular_L | -3.3768 | 0.0007 |
|  |  | Temporal_Mid_L | Temporal_Mid_L | -3.4844 | 0.0005 |
|  | AUD | Occipital_Mid_L | Temporal_Sup_R | -3.5652 | 0.0004 |
|  |  | Occipital_Mid_L | Temporal_Sup_L | -3.3768 | 0.0007 |
|  |  | Occipital_Mid_R | Temporal_Sup_R | -3.2692 | 0.0011 |
|  |  | Occipital_Mid_R | Rolandic_Oper_L | -3.3768 | 0.0007 |
|  |  | Occipital_Mid_R | undefined | -3.3230 | 0.0009 |
|  |  | Temporal_Pole_Mid_R | Temporal_Sup_L | -3.3230 | 0.0009 |
|  |  | Temporal_Pole_Mid_R | Postcentral_R | -3.5921 | 0.0003 |
|  |  | Precuneus_L | Rolandic_Oper_L | -3.2423 | 0.0012 |
|  |  | Precuneus_L | SupraMarginal_L | -3.4575 | 0.0005 |
|  |  | Precuneus_R | Temporal_Sup_L | -3.7266 | 0.0002 |
|  |  | Precuneus_R | undefined | -3.2961 | 0.0010 |
|  |  | Cingulum_Mid_L | Postcentral_R | -3.1616 | 0.0016 |
|  |  | Precuneus_R | Temporal_Sup_R | -3.3499 | 0.0008 |
|  |  | Precuneus_R | Temporal_Sup_L | -3.5921 | 0.0003 |
|  |  | Precuneus_R | undefined | -3.3768 | 0.0007 |
|  |  | Frontal_Sup_R | undefined | -3.6190 | 0.0003 |
|  |  | Temporal_Mid_R | Temporal_Sup_R | -3.2961 | 0.0010 |
|  |  | Temporal_Mid_L | Temporal_Sup_R | -3.1885 | 0.0014 |
|  |  | Temporal_Mid_L | Temporal_Sup_L | -3.5383 | 0.0004 |
|  |  | Temporal_Mid_L | Temporal_Sup_L | -3.2692 | 0.0011 |
|  |  | Temporal_Mid_L | SupraMarginal_L | -3.4844 | 0.0005 |
|  |  | Temporal_Mid_L | Rolandic_Oper_L | -3.5383 | 0.0004 |
|  |  | Temporal_Mid_L | Rolandic_Oper_L | -3.3499 | 0.0008 |
|  |  | Temporal_Mid_L | Temporal_Sup_L | -3.4306 | 0.0006 |
|  |  | Temporal_Mid_L | Temporal_Sup_L | -3.2692 | 0.0011 |
|  |  | Temporal_Mid_R | Rolandic_Oper_L | -3.9688 | 0.0001 |
|  |  | Temporal_Mid_R | Temporal_Sup_L | -3.3499 | 0.0008 |
|  |  | Temporal_Mid_R | Temporal_Sup_L | -3.4306 | 0.0006 |
|  |  | Temporal_Mid_R | Rolandic_Oper_L | -3.4037 | 0.0007 |
|  |  | Fusiform_R | Temporal_Sup_R | -3.2423 | 0.0012 |
|  |  | Temporal_Mid_L | Rolandic_Oper_L | -3.5114 | 0.0004 |
|  | CON | Occipital_Mid_L | Frontal_Sup_R | -3.3499 | 0.0008 |
|  |  | Occipital_Mid_L | Cingulum_Mid_L | -3.1616 | 0.0016 |
|  |  | Temporal_Mid_L | Frontal_Sup_R | -3.6190 | 0.0003 |
|  |  | Temporal_Mid_L | Cingulum_Mid_L | -3.6459 | 0.0003 |
|  |  | Temporal_Pole_Mid_L | Supp_Motor_Area_R | -3.3768 | 0.0007 |
|  |  | Angular_L | Frontal_Sup_R | -3.4575 | 0.0005 |
|  |  | Precuneus_L | Frontal_Sup_R | -3.5652 | 0.0004 |
|  |  | Precuneus_R | SupraMarginal_R | -3.1885 | 0.0014 |
|  |  | Temporal_Mid_L | Frontal_Sup_R | -3.6190 | 0.0003 |
|  |  | Temporal_Mid_L | Cingulum_Mid_L | -3.3230 | 0.0009 |
|  |  | Temporal_Mid_R | Frontal_Sup_R | -3.1885 | 0.0014 |
|  |  | ParaHippocampal_L | Temporal_Pole_Sup_L | -3.6190 | 0.0003 |
|  |  | Temporal_Mid_L | Frontal_Sup_R | -3.4844 | 0.0005 |
|  |  | Temporal_Mid_L | Supp_Motor_Area_R | -3.8073 | 0.0001 |
| VIN | SMN | Lingual_R | Precuneus_L | -3.2961 | 0.0010 |
|  |  | Occipital_Mid_R | Precuneus_L | -4.0764 | 0.0000 |
|  |  | Occipital_Mid_R | Postcentral_R | -3.9150 | 0.0001 |
|  |  | Occipital_Mid_R | Postcentral_L | -3.6459 | 0.0003 |
|  |  | Occipital_Mid_R | Postcentral_R | -3.8611 | 0.0001 |
|  |  | Occipital_Mid_R | Postcentral_L | -3.4037 | 0.0007 |
|  |  | Lingual_R | Parietal_Inf_L | -3.9688 | 0.0001 |
|  |  | Lingual_R | Postcentral_R | -3.2423 | 0.0012 |
|  |  | Lingual_R | Postcentral_R | -3.4844 | 0.0005 |
|  |  | Fusiform_R | Precentral_R | -3.2961 | 0.0010 |
|  |  | Lingual_L | Precuneus_L | -3.1885 | 0.0014 |
|  |  | Calcarine_L | Parietal_Inf_L | -3.3499 | 0.0008 |
|  |  | Occipital_Mid_R | Precuneus_L | -3.1616 | 0.0016 |
|  |  | Occipital_Mid_R | Parietal_Inf_L | -3.1885 | 0.0014 |
|  |  | Cuneus_R | Postcentral_L | -3.6997 | 0.0002 |
|  |  | Cuneus_R | Supp_Motor_Area_R | -3.3499 | 0.0008 |
|  |  | Lingual_L | Precuneus_L | -3.2423 | 0.0012 |
|  |  | Cuneus_R | Precentral_R | -3.4844 | 0.0005 |
|  |  | Cuneus_R | Postcentral_R | -3.4844 | 0.0005 |
|  |  | Cuneus_R | Supp_Motor_Area_R | -3.3230 | 0.0009 |
|  |  | Cuneus_L | Postcentral_L | -3.7266 | 0.0002 |
|  |  | Cuneus_L | Precuneus_L | -3.1885 | 0.0014 |
|  |  | Cuneus_L | Supp_Motor_Area_R | -3.1616 | 0.0016 |
|  |  | Cuneus_L | Supp_Motor_Area_R | -3.5383 | 0.0004 |
|  |  | Cuneus_L | Precentral_R | -3.3499 | 0.0008 |
|  | VIN | Lingual_L | Occipital_Mid_R | -3.1616 | 0.0016 |
|  |  | Cuneus_R | Occipital_Mid_R | -3.3499 | 0.0008 |
|  |  | Cuneus_L | Occipital_Mid_R | -3.2423 | 0.0012 |
|  |  | Cuneus_L | Occipital_Mid_R | -3.6190 | 0.0003 |
|  |  | Cuneus_L | Occipital_Mid_L | -3.3499 | 0.0008 |
|  |  | Cuneus_L | Occipital_Mid_R | -3.2154 | 0.0013 |
|  |  | Cuneus_L | Cuneus_R | -3.9150 | 0.0001 |
|  |  | Cuneus_L | Occipital_Sup_R | -3.3768 | 0.0007 |
|  |  | Cuneus_L | Cuneus_R | -3.4037 | 0.0007 |
|  |  | Cuneus_L | Cuneus_L | -3.5921 | 0.0003 |
|  | DMN | Lingual_R | Occipital_Mid_L | -3.1616 | 0.0016 |
|  |  | Lingual_R | Occipital_Mid_R | -3.2154 | 0.0013 |
|  |  | Occipital_Mid_R | Occipital_Mid_L | -3.1885 | 0.0014 |
|  |  | Calcarine_R | Occipital_Mid_R | -3.4575 | 0.0005 |
|  |  | Calcarine_L | Occipital_Mid_L | -3.2154 | 0.0013 |
|  |  | Calcarine_L | Occipital_Mid_R | -3.7804 | 0.0002 |
|  |  | Calcarine_L | Fusiform_R | -3.6728 | 0.0002 |
|  |  | Occipital_Mid_L | Occipital_Mid_L | -3.7804 | 0.0002 |
|  |  | Lingual_R | Occipital_Mid_L | -3.4037 | 0.0007 |
|  |  | Lingual_R | Occipital_Mid_R | -3.7804 | 0.0002 |
|  |  | Lingual_R | Temporal_Mid_R | -3.1616 | 0.0016 |
|  |  | Occipital_Mid_L | Occipital_Mid_L | -3.4037 | 0.0007 |
|  |  | Lingual_L | Occipital_Mid_L | -3.3499 | 0.0008 |
|  |  | Calcarine_L | Occipital_Mid_R | -3.9150 | 0.0001 |
|  |  | Calcarine_L | Temporal_Mid_R | -3.2423 | 0.0012 |
|  |  | Calcarine_L | Temporal_Mid_R | -3.4575 | 0.0005 |
|  |  | Occipital_Sup_L | Occipital_Mid_L | -3.1616 | 0.0016 |
|  |  | Occipital_Sup_L | Temporal_Mid_L | -3.1885 | 0.0014 |
|  |  | Occipital_Sup_L | Temporal_Mid_L | -3.1885 | 0.0014 |
|  |  | Cuneus_R | Fusiform_R | -3.2423 | 0.0012 |
|  |  | Occipital_Mid_R | Cingulum_Mid_L | -3.2154 | 0.0013 |
|  |  | Occipital_Mid_R | Frontal_Med_Orb_R | -3.5383 | 0.0004 |
|  |  | Cuneus_R | Occipital_Mid_L | -3.3230 | 0.0009 |
|  |  | Cuneus_R | Precuneus_R | -3.4575 | 0.0005 |
|  |  | Cuneus_R | Temporal_Mid_L | -3.3230 | 0.0009 |
|  |  | Cuneus_R | Fusiform_R | -3.1616 | 0.0016 |
|  |  | Cuneus_R | Fusiform_L | -3.6190 | 0.0003 |
|  |  | Lingual_L | Occipital_Mid_R | -3.2961 | 0.0010 |
|  |  | Occipital_Sup_R | Occipital_Mid_L | -3.5383 | 0.0004 |
|  |  | Occipital_Sup_R | Temporal_Mid_L | -3.4306 | 0.0006 |
|  |  | Cuneus_R | Occipital_Mid_L | -3.2423 | 0.0012 |
|  |  | Cuneus_R | Frontal_Sup_L | -3.1616 | 0.0016 |
|  |  | Cuneus_R | Temporal_Mid_L | -3.6728 | 0.0002 |
|  |  | Cuneus_R | Temporal_Mid_L | -3.3230 | 0.0009 |
|  |  | Cuneus_R | ParaHippocampal_L | -3.2423 | 0.0012 |
|  |  | Cuneus_R | Temporal_Mid_L | -3.6997 | 0.0002 |
|  |  | Occipital_Mid_L | Occipital_Mid_L | -3.2961 | 0.0010 |
|  |  | Occipital_Mid_L | Precuneus_R | -3.1885 | 0.0014 |
|  |  | Occipital_Mid_L | Cingulum_Mid_L | -3.2154 | 0.0013 |
|  |  | Occipital_Mid_L | Cingulum_Ant_L | -3.4037 | 0.0007 |
|  |  | Cuneus_L | Occipital_Mid_L | -4.3724 | 0.0000 |
|  |  | Cuneus_L | Occipital_Mid_R | -3.2692 | 0.0011 |
|  |  | Cuneus_L | Frontal_Sup_L | -3.8611 | 0.0001 |
|  |  | Cuneus_L | Temporal_Mid_L | -3.7266 | 0.0002 |
|  |  | Cuneus_L | Temporal_Mid_L | -3.7535 | 0.0002 |
|  |  | Cuneus_L | Temporal_Mid_L | -3.3768 | 0.0007 |
|  |  | Cuneus_L | Temporal_Mid_R | -3.4844 | 0.0005 |
|  |  | Cuneus_L | Fusiform_R | -3.5652 | 0.0004 |
|  |  | Cuneus_L | Occipital_Mid_L | -3.2692 | 0.0011 |
|  |  | Cuneus_L | ParaHippocampal_L | -3.9957 | 0.0001 |
|  |  | Cuneus_L | Temporal_Mid_L | -3.4575 | 0.0005 |
|  |  | Occipital_Mid_L | Angular_R | -3.5383 | 0.0004 |
|  |  | Occipital_Mid_L | Frontal_Sup_Medial_L | -3.2692 | 0.0011 |
|  |  | Calcarine_R | Occipital_Mid_R | -3.2692 | 0.0011 |
|  |  | Fusiform_L | Angular_R | -3.1885 | 0.0014 |
|  |  | Occipital_Mid_R | Temporal_Mid_L | -3.2961 | 0.0010 |
|  | AUD | Lingual_R | Postcentral_R | -3.2692 | 0.0011 |
|  |  | Lingual_L | Temporal_Sup_L | -3.3768 | 0.0007 |
|  |  | Lingual_L | SupraMarginal_L | -3.9957 | 0.0001 |
|  |  | Calcarine_L | Temporal_Sup_L | -3.2961 | 0.0010 |
|  |  | Cuneus_R | Postcentral_R | -3.2154 | 0.0013 |
|  |  | Cuneus_R | Temporal_Sup_R | -3.5652 | 0.0004 |
|  |  | Cuneus_R | Rolandic_Oper_L | -3.4844 | 0.0005 |
|  |  | Cuneus_R | Temporal_Sup_L | -3.7535 | 0.0002 |
|  |  | Cuneus_R | Postcentral_R | -3.5383 | 0.0004 |
|  |  | Cuneus_R | undefined | -3.6190 | 0.0003 |
|  |  | Occipital_Sup_R | Temporal_Sup_L | -3.3768 | 0.0007 |
|  |  | Cuneus_R | Rolandic_Oper_L | -3.2423 | 0.0012 |
|  |  | Cuneus_R | Temporal_Sup_L | -3.8880 | 0.0001 |
|  |  | Cuneus_R | Postcentral_R | -3.4306 | 0.0006 |
|  |  | Cuneus_L | Rolandic_Oper_L | -3.6728 | 0.0002 |
|  | CON | Lingual_R | SupraMarginal_R | -3.5114 | 0.0004 |
|  |  | Lingual_R | undefined | -3.6459 | 0.0003 |
|  |  | Lingual_L | SupraMarginal_R | -3.3768 | 0.0007 |
|  |  | Cuneus_L | Temporal_Pole_Sup_L | -3.2154 | 0.0013 |
| FPN | SMN | Precentral_L | Precuneus_L | -3.4844 | 0.0005 |
|  |  | Precentral_L | Supp_Motor_Area_L | -3.6728 | 0.0002 |
|  |  | Precentral_L | Parietal_Inf_L | -3.8342 | 0.0001 |
|  |  | Precentral_L | Precentral_L | -3.2154 | 0.0013 |
|  |  | Precentral_L | Postcentral_R | -3.5652 | 0.0004 |
|  |  | Precentral_L | undefined | -3.9150 | 0.0001 |
|  |  | Precentral_L | Postcentral_L | -3.4844 | 0.0005 |
|  |  | Precentral_L | Postcentral_L | -3.1885 | 0.0014 |
|  |  | Precentral_L | Postcentral_R | -3.5383 | 0.0004 |
|  |  | Precentral_L | Postcentral_R | -3.2961 | 0.0010 |
|  |  | Frontal_Inf_Tri_L | Precuneus_L | -3.9688 | 0.0001 |
|  |  | Frontal_Inf_Tri_L | Supp_Motor_Area_L | -4.0764 | 0.0000 |
|  |  | Frontal_Inf_Tri_L | Supp_Motor_Area_R | -3.8342 | 0.0001 |
|  |  | Frontal_Inf_Tri_L | Insula_R | -3.3768 | 0.0007 |
|  |  | Precentral_R | Supp_Motor_Area_R | -3.2961 | 0.0010 |
|  |  | Precentral_R | Postcentral_L | -3.1885 | 0.0014 |
|  |  | Parietal_Inf_L | Supp_Motor_Area_L | -3.1616 | 0.0016 |
|  |  | Frontal_Mid_Orb_L | Supp_Motor_Area_L | -3.5114 | 0.0004 |
|  |  | Frontal_Mid_Orb_L | Supp_Motor_Area_R | -3.1885 | 0.0014 |
|  |  | Frontal_Inf_Tri_L | Supp_Motor_Area_L | -3.7535 | 0.0002 |
|  | VIN | Frontal_Mid_Orb_R | Cuneus_R | -3.1616 | 0.0016 |
|  |  | Parietal_Inf_R | Occipital_Inf_L | -3.2154 | 0.0013 |
|  |  | Parietal_Inf_R | Occipital_Mid_L | -3.7266 | 0.0002 |
|  |  | Parietal_Inf_R | Occipital_Mid_L | -3.2961 | 0.0010 |
|  |  | Parietal_Inf_R | Fusiform_L | -3.3230 | 0.0009 |
|  |  | Parietal_Inf_R | Fusiform_L | -3.5652 | 0.0004 |
|  |  | Frontal_Mid_R | Occipital_Mid_L | -3.4575 | 0.0005 |
|  |  | Frontal_Mid_L | Occipital_Mid_L | -3.3230 | 0.0009 |
|  |  | Frontal_Mid_L | Occipital_Mid_L | -3.2154 | 0.0013 |
|  | DMN | Precentral_L | Occipital_Mid_L | -3.3230 | 0.0009 |
|  |  | Precentral_L | Precuneus_R | -3.2423 | 0.0012 |
|  |  | Precentral_L | Temporal_Mid_R | -3.3768 | 0.0007 |
|  |  | Frontal_Inf_Tri_L | ParaHippocampal_L | -3.3230 | 0.0009 |
|  |  | Precentral_L | Precuneus_L | -3.5114 | 0.0004 |
|  |  | Frontal_Mid_Orb_L | Precuneus_L | -3.2423 | 0.0012 |
|  |  | Frontal_Mid_Orb_L | Frontal_Med_Orb_R | -3.2423 | 0.0012 |
|  | AUD | Precentral_L | Temporal_Sup_R | -3.2154 | 0.0013 |
|  |  | Precentral_L | Temporal_Sup_L | -3.3230 | 0.0009 |
|  |  | Precentral_L | SupraMarginal_L | -3.3768 | 0.0007 |
|  |  | Precentral_L | Rolandic_Oper_R | -3.8342 | 0.0001 |
|  |  | Precentral_L | Postcentral_R | -3.5652 | 0.0004 |
|  |  | Frontal_Inf_Tri_R | SupraMarginal_L | -3.6997 | 0.0002 |
|  |  | Frontal_Inf_Tri_R | undefined | -3.5383 | 0.0004 |
|  |  | Frontal_Inf_Tri_L | Temporal_Sup_R | -3.8342 | 0.0001 |
|  |  | Frontal_Inf_Tri_L | Rolandic_Oper_R | -3.1885 | 0.0014 |
|  |  | Frontal_Inf_Tri_L | SupraMarginal_L | -3.6459 | 0.0003 |
|  |  | Frontal_Inf_Tri_L | SupraMarginal_L | -3.6459 | 0.0003 |
|  |  | Frontal_Mid_Orb_R | Temporal_Sup_R | -3.4844 | 0.0005 |
|  |  | Precentral_R | SupraMarginal_L | -3.7804 | 0.0002 |
|  |  | Precentral_R | undefined | -3.3768 | 0.0007 |
|  |  | Angular_R | undefined | -3.2423 | 0.0012 |
|  |  | Frontal_Mid_Orb_L | SupraMarginal_L | -3.2154 | 0.0013 |
|  | CON | Frontal_Inf_Tri_L | Rolandic_Oper_L | -3.1885 | 0.0014 |
|  |  | Frontal_Inf_Tri_L | Temporal_Pole_Sup_L | -3.2692 | 0.0011 |
|  |  | Precentral_R | undefined | -3.2692 | 0.0011 |
|  |  | Parietal_Inf_L | undefined | -3.2423 | 0.0012 |
|  |  | Parietal_Inf_L | Temporal_Pole_Sup_L | -3.2961 | 0.0010 |
|  |  | Parietal_Inf_L | undefined | -3.7266 | 0.0002 |
|  |  | Angular_R | SupraMarginal_R | -3.4844 | 0.0005 |
|  |  | Frontal_Inf_Tri_L | Frontal_Sup_R | -3.2154 | 0.0013 |
|  |  | Frontal_Sup_Medial_L | Supp_Motor_Area_R | -3.2961 | 0.0010 |
|  |  | Frontal_Sup_Medial_L | undefined | -3.1885 | 0.0014 |
| SAN | SMN | Cingulum_Mid_R | Precentral_R | -3.1885 | 0.0014 |
|  |  | Cingulum_Mid_R | Postcentral_L | -3.3499 | 0.0008 |
|  |  | Cingulum_Mid_R | undefined | -3.9419 | 0.0001 |
|  |  | Cingulum_Mid_R | Precentral_R | -3.5114 | 0.0004 |
|  |  | Cingulum_Mid_R | Postcentral_R | -3.5921 | 0.0003 |
|  |  | Precentral_R | Supp_Motor_Area_R | -3.8880 | 0.0001 |
|  |  | Precentral_R | Postcentral_R | -3.1616 | 0.0016 |
|  |  | Insula_L | Insula_R | -3.6190 | 0.0003 |
|  | SAN | Supp_Motor_Area_L | Insula_L | -3.1616 | 0.0016 |
|  | FPN | Cingulum_Mid_R | Precentral_L | -3.5383 | 0.0004 |
|  |  | Cingulum_Mid_R | Frontal_Mid_Orb_R | -3.1885 | 0.0014 |
|  |  | Cingulum_Mid_R | Precentral_R | -3.5383 | 0.0004 |
|  |  | Cingulum_Mid_R | Parietal_Inf_L | -3.5114 | 0.0004 |
|  |  | Cingulum_Mid_R | Angular_R | -3.9688 | 0.0001 |
|  |  | Cingulum_Mid_R | Frontal_Inf_Tri_L | -3.5921 | 0.0003 |
|  |  | Insula_L | Parietal_Inf_L | -3.1885 | 0.0014 |
|  |  | Insula_R | Parietal_Inf_L | -3.7535 | 0.0002 |
|  |  | Supp_Motor_Area_L | Parietal_Inf_L | -3.2961 | 0.0010 |
|  |  | Cingulum_Ant_L | Angular_R | -3.5114 | 0.0004 |
|  |  | Frontal_Mid_L | Frontal_Mid_Orb_R | -3.2692 | 0.0011 |
|  | VIN | Cingulum_Mid_R | Lingual_R | -3.5652 | 0.0004 |
|  |  | Cingulum_Mid_R | Occipital_Mid_R | -3.5652 | 0.0004 |
|  |  | Cingulum_Mid_R | Calcarine_R | -3.6190 | 0.0003 |
|  |  | Cingulum_Mid_R | Calcarine_L | -4.0495 | 0.0001 |
|  |  | Cingulum_Mid_R | Lingual_R | -3.5383 | 0.0004 |
|  |  | Cingulum_Mid_R | Fusiform_R | -3.4037 | 0.0007 |
|  |  | Cingulum_Mid_R | Lingual_L | -3.4844 | 0.0005 |
|  |  | Cingulum_Mid_R | Calcarine_L | -3.5921 | 0.0003 |
|  |  | Cingulum_Mid_R | Cuneus_R | -3.7535 | 0.0002 |
|  |  | Cingulum_Mid_R | Cuneus_R | -3.8073 | 0.0001 |
|  |  | Cingulum_Mid_R | Cuneus_L | -3.4844 | 0.0005 |
|  |  | Parietal_Inf_R | Occipital_Mid_L | -3.5383 | 0.0004 |
|  |  | Parietal_Inf_R | Occipital_Mid_L | -3.4844 | 0.0005 |
|  |  | Frontal_Inf_Tri_R | Calcarine_R | -4.0226 | 0.0001 |
|  |  | Frontal_Inf_Tri_R | Lingual_R | -3.1885 | 0.0014 |
|  |  | Frontal_Inf_Tri_R | Calcarine_L | -3.1885 | 0.0014 |
|  |  | Frontal_Inf_Tri_R | Cuneus_R | -3.5383 | 0.0004 |
|  |  | Frontal_Inf_Tri_R | Calcarine_R | -3.5921 | 0.0003 |
|  |  | Insula_L | Calcarine_R | -3.6728 | 0.0002 |
|  |  | Insula_L | Calcarine_L | -3.4306 | 0.0006 |
|  |  | Insula_R | Calcarine_R | -4.2647 | 0.0000 |
|  |  | Insula_R | Calcarine_L | -3.8880 | 0.0001 |
|  |  | Insula_R | Calcarine_R | -3.1885 | 0.0014 |
|  |  | undefined | Calcarine_L | -3.3499 | 0.0008 |
|  |  | Cingulum_Ant_L | Occipital_Mid_L | -3.4306 | 0.0006 |
|  |  | Frontal_Mid_R | Occipital_Mid_L | -3.2154 | 0.0013 |
|  |  | Frontal_Mid_R | Occipital_Mid_L | -3.2692 | 0.0011 |
|  | DMN | Cingulum_Mid_R | Occipital_Mid_L | -3.2423 | 0.0012 |
|  |  | Cingulum_Mid_R | Occipital_Mid_R | -4.0764 | 0.0000 |
|  |  | Cingulum_Mid_R | Precuneus_L | -3.2423 | 0.0012 |
|  |  | Cingulum_Mid_R | Temporal_Mid_R | -3.4844 | 0.0005 |
|  |  | Cingulum_Mid_R | Fusiform_R | -3.3499 | 0.0008 |
|  |  | Cingulum_Mid_R | Temporal_Mid_L | -3.4575 | 0.0005 |
|  |  | Precentral_R | Precuneus_R | -3.2961 | 0.0010 |
|  |  | Precentral_R | Temporal_Mid_L | -3.3499 | 0.0008 |
|  |  | Insula_L | Precuneus_R | -3.2961 | 0.0010 |
|  |  | Frontal_Inf_Orb_R | Cingulum_Ant_R | -3.1885 | 0.0014 |
|  |  | undefined | Precuneus_L | -3.1616 | 0.0016 |
|  |  | Cingulum_Mid_R | Cingulum_Ant_R | -3.2423 | 0.0012 |
|  |  | Cingulum_Ant_R | Rectus_R | -3.1885 | 0.0014 |
|  |  | Cingulum_Ant_R | Occipital_Mid_R | -3.2961 | 0.0010 |
|  |  | Cingulum_Ant_R | Cingulum_Ant_R | -3.2154 | 0.0013 |
|  | AUD | Cingulum_Mid_R | Rolandic_Oper_L | -3.5921 | 0.0003 |
|  |  | Cingulum_Mid_R | Temporal_Sup_L | -3.7266 | 0.0002 |
|  |  | Cingulum_Mid_R | Postcentral_R | -3.2154 | 0.0013 |
|  |  | Precentral_R | Temporal_Sup_R | -3.5921 | 0.0003 |
|  |  | Frontal_Inf_Tri_R | undefined | -3.9419 | 0.0001 |
|  |  | Frontal_Inf_Tri_R | Temporal_Sup_L | -3.2423 | 0.0012 |
|  |  | Frontal_Inf_Orb_R | Rolandic_Oper_R | -3.3499 | 0.0008 |
|  | CON | Precentral_R | Rolandic_Oper_L | -3.1885 | 0.0014 |
|  |  | Frontal_Inf_Tri_R | undefined | -3.2154 | 0.0013 |
| SUB | FPN | Putamen_L | Parietal_Inf_L | -3.6728 | 0.0002 |
|  |  | Putamen_R | Frontal_Mid_Orb_R | -3.3499 | 0.0008 |
|  |  | Putamen_R | Frontal_Mid_Orb_R | -3.8342 | 0.0001 |
|  |  | undefined | Frontal_Mid_Orb_R | -3.2154 | 0.0013 |
|  | VIN | Thalamus_L | Fusiform_R | -3.2961 | 0.0010 |
|  |  | Thalamus_R | Calcarine_R | -3.5921 | 0.0003 |
|  |  | Thalamus_R | Calcarine_R | -3.6459 | 0.0003 |
|  |  | undefined | Calcarine_R | -3.2961 | 0.0010 |
|  | DMN | Putamen_L | Frontal_Sup_Medial_L | -3.2692 | 0.0011 |
|  |  | Putamen_R | Frontal_Mid_L | -3.2692 | 0.0011 |
|  |  | Putamen_R | Frontal_Sup_L | -3.2692 | 0.0011 |
| VAN | SMN | Temporal_Mid_L | undefined | -3.6728 | 0.0002 |
|  |  | Temporal_Mid_L | Precentral_R | -3.2423 | 0.0012 |
|  |  | Temporal_Mid_L | undefined | -3.5652 | 0.0004 |
|  |  | Temporal_Mid_L | Precentral_R | -3.7266 | 0.0002 |
|  |  | Temporal_Mid_L | Precentral_R | -3.8611 | 0.0001 |
|  |  | Temporal_Mid_L | Postcentral_R | -3.2423 | 0.0012 |
|  |  | Temporal_Sup_L | Paracentral_Lobule_L | -3.4844 | 0.0005 |
|  |  | Temporal_Sup_L | Precentral_R | -3.1616 | 0.0016 |
|  |  | Temporal_Sup_L | Precentral_R | -3.9688 | 0.0001 |
|  |  | Temporal_Sup_L | Postcentral_L | -3.7535 | 0.0002 |
|  |  | Temporal_Sup_L | Precentral_R | -3.7535 | 0.0002 |
|  |  | Temporal_Sup_L | Supp_Motor_Area_R | -3.7535 | 0.0002 |
|  |  | Temporal_Sup_L | Postcentral_R | -3.3768 | 0.0007 |
|  |  | Temporal_Sup_R | Supp_Motor_Area_R | -3.3499 | 0.0008 |
|  |  | Temporal_Sup_R | Precentral_R | -3.4037 | 0.0007 |
|  |  | Temporal_Sup_R | undefined | -3.7535 | 0.0002 |
|  |  | Temporal_Sup_R | Supp_Motor_Area_R | -3.2423 | 0.0012 |
|  |  | Temporal_Sup_R | Postcentral_R | -3.7804 | 0.0002 |
|  |  | Temporal_Mid_R | Precuneus_L | -3.5383 | 0.0004 |
|  |  | Temporal_Mid_R | Supp_Motor_Area_R | -3.4306 | 0.0006 |
|  |  | Temporal_Mid_R | undefined | -3.7804 | 0.0002 |
|  |  | Temporal_Mid_R | undefined | -3.3230 | 0.0009 |
|  |  | Frontal_Inf_Tri_L | Precuneus_L | -3.1885 | 0.0014 |
|  |  | Frontal_Inf_Tri_L | undefined | -3.2154 | 0.0013 |
|  | VAN | Temporal_Sup_L | Temporal_Mid_L | -3.5114 | 0.0004 |
|  |  | Temporal_Sup_R | Temporal_Mid_L | -3.9688 | 0.0001 |
|  |  | Temporal_Sup_R | Temporal_Sup_L | -3.6728 | 0.0002 |
|  |  | Temporal_Mid_R | Temporal_Sup_L | -3.5652 | 0.0004 |
|  |  | Frontal_Inf_Tri_L | Temporal_Sup_L | -3.3499 | 0.0008 |
|  | SAN | Temporal_Mid_L | Cingulum_Mid_R | -3.4037 | 0.0007 |
|  |  | Temporal_Sup_L | Frontal_Inf_Orb_R | -3.4575 | 0.0005 |
|  |  | Temporal_Sup_R | Cingulum_Mid_R | -3.3768 | 0.0007 |
|  |  | Temporal_Sup_R | Frontal_Inf_Tri_R | -3.1885 | 0.0014 |
|  |  | Temporal_Sup_R | Frontal_Inf_Orb_R | -3.4306 | 0.0006 |
|  |  | Temporal_Mid_R | Cingulum_Mid_R | -3.5114 | 0.0004 |
|  |  | Temporal_Mid_R | Frontal_Inf_Orb_R | -3.1885 | 0.0014 |
|  | FPN | Temporal_Sup_R | Frontal_Mid_Orb_R | -3.1616 | 0.0016 |
|  |  | Temporal_Mid_L | Precentral_L | -3.4037 | 0.0007 |
|  |  | Temporal_Sup_L | Precentral_L | -4.0764 | 0.0000 |
|  |  | Temporal_Sup_R | Precentral_L | -3.8342 | 0.0001 |
|  |  | Temporal_Sup_R | Frontal_Inf_Tri_L | -3.8342 | 0.0001 |
|  |  | Temporal_Sup_R | Frontal_Mid_Orb_R | -3.7266 | 0.0002 |
|  | VIN | Temporal_Mid_L | Lingual_R | -4.0764 | 0.0000 |
|  |  | Temporal_Mid_L | Lingual_L | -3.5114 | 0.0004 |
|  |  | Temporal_Mid_L | Occipital_Sup_L | -3.6190 | 0.0003 |
|  |  | Temporal_Mid_L | Occipital_Mid_R | -3.3768 | 0.0007 |
|  |  | Temporal_Mid_L | Cuneus_R | -4.0226 | 0.0001 |
|  |  | Temporal_Mid_L | Lingual_L | -3.6190 | 0.0003 |
|  |  | Temporal_Mid_L | Occipital_Sup_R | -3.7266 | 0.0002 |
|  |  | Temporal_Mid_L | Cuneus_R | -3.3499 | 0.0008 |
|  |  | Temporal_Mid_L | Cuneus_L | -4.0226 | 0.0001 |
|  |  | Temporal_Mid_L | Cuneus_L | -3.5114 | 0.0004 |
|  |  | Temporal_Sup_L | Lingual_L | -3.4037 | 0.0007 |
|  |  | Temporal_Sup_L | Cuneus_R | -3.2423 | 0.0012 |
|  |  | Temporal_Sup_L | Lingual_L | -3.1885 | 0.0014 |
|  |  | Temporal_Sup_L | Cuneus_R | -3.4575 | 0.0005 |
|  |  | Temporal_Sup_L | Cuneus_L | -3.5383 | 0.0004 |
|  |  | Temporal_Sup_R | Lingual_R | -3.3230 | 0.0009 |
|  |  | Temporal_Sup_R | Lingual_L | -3.9688 | 0.0001 |
|  |  | Temporal_Sup_R | Cuneus_R | -3.3499 | 0.0008 |
|  |  | Temporal_Sup_R | Occipital_Sup_R | -3.3499 | 0.0008 |
|  |  | Temporal_Sup_R | Cuneus_R | -3.3499 | 0.0008 |
|  |  | Temporal_Sup_R | Cuneus_L | -3.4575 | 0.0005 |
|  |  | Temporal_Sup_R | Cuneus_L | -3.8611 | 0.0001 |
|  |  | Temporal_Mid_R | Lingual_R | -3.2154 | 0.0013 |
|  |  | Temporal_Mid_R | Lingual_L | -3.3768 | 0.0007 |
|  |  | Temporal_Mid_R | Calcarine_L | -3.3230 | 0.0009 |
|  |  | Temporal_Mid_R | Cuneus_R | -3.7535 | 0.0002 |
|  |  | Temporal_Mid_R | Lingual_L | -3.6190 | 0.0003 |
|  |  | Temporal_Mid_R | Cuneus_R | -3.6728 | 0.0002 |
|  |  | Temporal_Mid_R | Cuneus_L | -3.4306 | 0.0006 |
|  |  | Temporal_Mid_R | Cuneus_L | -3.4306 | 0.0006 |
|  | DMN | Temporal_Sup_R | Occipital_Mid_L | -3.2692 | 0.0011 |
|  |  | Temporal_Sup_R | Precuneus_R | -3.3768 | 0.0007 |
|  |  | Temporal_Mid_L | Temporal_Mid_L | -3.8611 | 0.0001 |
|  |  | Temporal_Mid_L | Occipital_Mid_R | -3.5383 | 0.0004 |
|  |  | Temporal_Mid_L | Precuneus_L | -3.9688 | 0.0001 |
|  |  | Temporal_Mid_L | Precuneus_L | -4.0764 | 0.0000 |
|  |  | Temporal_Mid_L | Precuneus_R | -3.8880 | 0.0001 |
|  |  | Temporal_Mid_L | Precuneus_R | -4.0764 | 0.0000 |
|  |  | Temporal_Mid_L | Frontal_Med_Orb_L | -3.8342 | 0.0001 |
|  |  | Temporal_Mid_L | Temporal_Mid_L | -3.7804 | 0.0002 |
|  |  | Temporal_Mid_L | Temporal_Mid_R | -3.5652 | 0.0004 |
|  |  | Temporal_Mid_L | ParaHippocampal_L | -3.6459 | 0.0003 |
|  |  | Temporal_Mid_L | Fusiform_R | -3.4037 | 0.0007 |
|  |  | Temporal_Mid_L | Temporal_Mid_L | -3.6190 | 0.0003 |
|  |  | Temporal_Sup_L | Temporal_Mid_L | -3.6190 | 0.0003 |
|  |  | Temporal_Sup_L | Temporal_Pole_Mid_R | -3.5114 | 0.0004 |
|  |  | Temporal_Sup_L | Temporal_Mid_L | -3.5114 | 0.0004 |
|  |  | Temporal_Sup_L | Angular_L | -3.5114 | 0.0004 |
|  |  | Temporal_Sup_L | Angular_L | -3.2154 | 0.0013 |
|  |  | Temporal_Sup_L | Precuneus_L | -3.4037 | 0.0007 |
|  |  | Temporal_Sup_L | Cingulum_Mid_R | -3.2154 | 0.0013 |
|  |  | Temporal_Sup_L | Precuneus_R | -3.3230 | 0.0009 |
|  |  | Temporal_Sup_L | Frontal_Med_Orb_L | -3.2154 | 0.0013 |
|  |  | Temporal_Sup_L | Frontal_Sup_Medial_L | -3.3499 | 0.0008 |
|  |  | Temporal_Sup_L | Temporal_Mid_R | -3.6459 | 0.0003 |
|  |  | Temporal_Sup_L | Temporal_Mid_L | -4.2109 | 0.0000 |
|  |  | Temporal_Sup_L | Temporal_Mid_L | -3.6728 | 0.0002 |
|  |  | Temporal_Sup_L | Temporal_Mid_L | -3.4306 | 0.0006 |
|  |  | Temporal_Sup_L | Temporal_Mid_R | -3.6459 | 0.0003 |
|  |  | Temporal_Sup_L | ParaHippocampal_L | -3.7266 | 0.0002 |
|  |  | Temporal_Sup_L | Fusiform_R | -3.6728 | 0.0002 |
|  |  | Temporal_Sup_L | Temporal_Mid_L | -3.2961 | 0.0010 |
|  |  | Temporal_Sup_R | Temporal_Mid_L | -3.6190 | 0.0003 |
|  |  | Temporal_Sup_R | Temporal_Mid_L | -3.8880 | 0.0001 |
|  |  | Temporal_Sup_R | Temporal_Mid_L | -3.4844 | 0.0005 |
|  |  | Temporal_Sup_R | Temporal_Mid_R | -3.8880 | 0.0001 |
|  |  | Temporal_Sup_R | Fusiform_R | -3.6459 | 0.0003 |
|  |  | Temporal_Mid_R | Precuneus_R | -3.2961 | 0.0010 |
|  |  | Temporal_Mid_R | Temporal_Mid_R | -3.2692 | 0.0011 |
|  |  | Temporal_Sup_R | Occipital_Mid_L | -3.4306 | 0.0006 |
|  |  | Temporal_Sup_R | Temporal_Mid_L | -4.3186 | 0.0000 |
|  |  | Temporal_Sup_R | Precuneus_L | -3.4306 | 0.0006 |
|  |  | Temporal_Sup_R | Precuneus_R | -3.4037 | 0.0007 |
|  |  | Temporal_Sup_R | Temporal_Mid_L | -3.6997 | 0.0002 |
|  |  | Temporal_Sup_R | Temporal_Mid_L | -3.8073 | 0.0001 |
|  |  | Temporal_Sup_R | Temporal_Mid_R | -3.2692 | 0.0011 |
|  |  | Temporal_Sup_R | ParaHippocampal_L | -3.3499 | 0.0008 |
|  |  | Temporal_Sup_R | Fusiform_R | -3.6728 | 0.0002 |
|  |  | Temporal_Sup_R | Temporal_Mid_L | -3.2423 | 0.0012 |
|  | AUD | Temporal_Mid_L | Temporal_Sup_R | -3.7535 | 0.0002 |
|  |  | Temporal_Mid_L | Rolandic_Oper_L | -3.5383 | 0.0004 |
|  |  | Temporal_Mid_L | Temporal_Sup_L | -3.6728 | 0.0002 |
|  |  | Temporal_Mid_L | Temporal_Sup_L | -3.9688 | 0.0001 |
|  |  | Temporal_Mid_L | Rolandic_Oper_R | -3.7804 | 0.0002 |
|  |  | Temporal_Sup_L | Temporal_Sup_L | -3.2423 | 0.0012 |
|  |  | Temporal_Sup_R | Temporal_Sup_R | -3.5652 | 0.0004 |
|  |  | Temporal_Sup_R | Temporal_Sup_L | -3.9957 | 0.0001 |
|  |  | Temporal_Sup_R | Rolandic_Oper_R | -3.3499 | 0.0008 |
|  |  | Temporal_Sup_R | Postcentral_R | -3.5383 | 0.0004 |
|  |  | Temporal_Mid_R | undefined | -3.5652 | 0.0004 |
|  |  | Temporal_Mid_R | Temporal_Sup_R | -4.0495 | 0.0001 |
|  |  | Temporal_Mid_R | Rolandic_Oper_L | -3.2692 | 0.0011 |
|  |  | Temporal_Mid_R | Temporal_Sup_L | -3.1616 | 0.0016 |
|  |  | Temporal_Mid_R | Rolandic_Oper_R | -3.2692 | 0.0011 |
|  |  | Temporal_Mid_R | SupraMarginal_L | -3.3499 | 0.0008 |
|  |  | Temporal_Mid_R | Rolandic_Oper_L | -3.6459 | 0.0003 |
|  |  | Temporal_Mid_R | undefined | -3.6190 | 0.0003 |
|  |  | Frontal_Inf_Tri_L | Rolandic_Oper_L | -3.1885 | 0.0014 |
|  | CON | Temporal_Sup_R | Frontal_Sup_R | -3.4844 | 0.0005 |
|  |  | Temporal_Sup_R | Temporal_Pole_Sup_L | -3.6190 | 0.0003 |
|  |  | Temporal_Mid_R | Supp_Motor_Area_R | -3.2154 | 0.0013 |
| DAN | SMN | Temporal_Mid_R | Precuneus_L | -3.3499 | 0.0008 |
|  |  | Temporal_Mid_R | Paracentral_Lobule_R | -3.1616 | 0.0016 |
|  |  | Occipital_Mid_L | Precuneus_L | -3.2961 | 0.0010 |
|  |  | Precentral_L | undefined | -3.4575 | 0.0005 |
|  |  | Precentral_L | Precentral_R | -3.2692 | 0.0011 |
|  |  | Precentral_L | Supp_Motor_Area_R | -3.1885 | 0.0014 |
|  |  | Precentral_L | Insula_R | -3.8342 | 0.0001 |
|  |  | Precentral_R | Supp_Motor_Area_L | -3.4037 | 0.0007 |
|  | DAN | Temporal_Mid_R | Temporal_Mid_L | -3.5114 | 0.0004 |
|  |  | Precentral_R | Precentral_L | -3.2423 | 0.0012 |
|  | VAN | Temporal_Mid_L | Temporal_Mid_L | -3.4306 | 0.0006 |
|  |  | Temporal_Mid_L | Temporal_Sup_R | -3.2692 | 0.0011 |
|  |  | Parietal_Sup_R | Temporal_Sup_R | -3.1616 | 0.0016 |
|  |  | Parietal_Sup_R | Temporal_Sup_R | -3.5652 | 0.0004 |
|  |  | Temporal_Mid_R | Temporal_Mid_L | -3.3230 | 0.0009 |
|  |  | Temporal_Mid_R | Temporal_Sup_R | -3.3499 | 0.0008 |
|  |  | Temporal_Mid_R | Frontal_Inf_Tri_L | -3.5114 | 0.0004 |
|  |  | Parietal_Sup_R | Temporal_Mid_R | -3.2154 | 0.0013 |
|  |  | Occipital_Mid_L | Temporal_Sup_R | -3.6190 | 0.0003 |
|  |  | Occipital_Mid_L | Temporal_Sup_R | -3.3499 | 0.0008 |
|  |  | Precentral_L | Temporal_Sup_R | -3.6997 | 0.0002 |
|  |  | Temporal_Inf_L | Temporal_Sup_L | -3.2692 | 0.0011 |
|  |  | Precentral_R | Temporal_Sup_R | -3.7535 | 0.0002 |
|  |  | Precentral_R | Temporal_Mid_R | -3.4306 | 0.0006 |
|  | SAN | Parietal_Sup_R | Parietal_Inf_R | -3.1616 | 0.0016 |
|  |  | Occipital_Mid_L | Frontal_Mid_L | -3.2692 | 0.0011 |
|  | FPN | Temporal_Mid_L | Precentral_L | -3.1616 | 0.0016 |
|  |  | Precentral_L | Precentral_L | -3.2423 | 0.0012 |
|  |  | Precentral_R | Precentral_L | -3.4844 | 0.0005 |
|  | VIN | Temporal_Mid_L | Occipital_Sup_R | -3.3768 | 0.0007 |
|  |  | Temporal_Mid_L | Cuneus_L | -3.2154 | 0.0013 |
|  |  | Temporal_Mid_R | Lingual_L | -3.1885 | 0.0014 |
|  |  | Temporal_Inf_L | Calcarine_R | -3.3230 | 0.0009 |
|  |  | Temporal_Inf_L | Lingual_L | -3.1885 | 0.0014 |
|  |  | Precentral_R | Lingual_L | -3.1616 | 0.0016 |
|  | DMN | Precuneus_R | Temporal_Mid_L | -3.2154 | 0.0013 |
|  |  | Temporal_Mid_L | Occipital_Mid_L | -3.7266 | 0.0002 |
|  |  | Temporal_Mid_L | Temporal_Mid_L | -4.2378 | 0.0000 |
|  |  | Temporal_Mid_L | Occipital_Mid_R | -3.5921 | 0.0003 |
|  |  | Temporal_Mid_L | Precuneus_L | -3.3768 | 0.0007 |
|  |  | Temporal_Mid_L | Cingulum_Mid_R | -3.3499 | 0.0008 |
|  |  | Temporal_Mid_L | Precuneus_R | -3.2423 | 0.0012 |
|  |  | Temporal_Mid_L | Frontal_Sup_L | -3.4844 | 0.0005 |
|  |  | Temporal_Mid_L | Temporal_Mid_L | -3.3499 | 0.0008 |
|  |  | Temporal_Mid_L | Temporal_Mid_R | -3.8073 | 0.0001 |
|  |  | Temporal_Mid_L | Fusiform_R | -3.4037 | 0.0007 |
|  |  | Temporal_Mid_L | Temporal_Mid_L | -3.6190 | 0.0003 |
|  |  | Parietal_Sup_R | Occipital_Mid_R | -3.9957 | 0.0001 |
|  |  | Temporal_Mid_R | Occipital_Mid_L | -3.3230 | 0.0009 |
|  |  | Temporal_Mid_R | Temporal_Mid_L | -3.3499 | 0.0008 |
|  |  | Temporal_Mid_R | Occipital_Mid_R | -3.2154 | 0.0013 |
|  |  | Temporal_Mid_R | Temporal_Mid_L | -3.4575 | 0.0005 |
|  |  | Temporal_Mid_R | Temporal_Mid_L | -3.4037 | 0.0007 |
|  |  | Temporal_Mid_R | Temporal_Mid_R | -3.4306 | 0.0006 |
|  |  | Temporal_Mid_R | ParaHippocampal_L | -3.5652 | 0.0004 |
|  |  | Temporal_Mid_R | Fusiform_L | -3.2423 | 0.0012 |
|  |  | Parietal_Sup_R | Occipital_Mid_R | -3.2961 | 0.0010 |
|  |  | Parietal_Inf_L | Occipital_Mid_R | -3.1616 | 0.0016 |
|  |  | Parietal_Inf_L | ParaHippocampal_L | -3.3499 | 0.0008 |
|  |  | Occipital_Mid_L | Occipital_Mid_L | -3.8073 | 0.0001 |
|  |  | Occipital_Mid_L | Occipital_Mid_R | -3.8611 | 0.0001 |
|  |  | Occipital_Mid_L | Angular_L | -3.1885 | 0.0014 |
|  |  | Occipital_Mid_L | Temporal_Mid_L | -3.4844 | 0.0005 |
|  |  | Parietal_Sup_L | Occipital_Mid_L | -3.1885 | 0.0014 |
|  |  | Parietal_Sup_L | Temporal_Mid_L | -3.3230 | 0.0009 |
|  |  | Parietal_Sup_L | Occipital_Mid_R | -3.3499 | 0.0008 |
|  |  | Precentral_R | Temporal_Pole_Mid_R | -3.4037 | 0.0007 |
|  |  | Precentral_R | Precuneus_L | -3.2961 | 0.0010 |
|  |  | Precentral_R | Temporal_Mid_L | -3.3230 | 0.0009 |
|  | AUD | Temporal_Mid_R | Postcentral_R | -3.6459 | 0.0003 |
|  |  | Precentral_L | Temporal_Sup_R | -3.6459 | 0.0003 |
|  |  | Precentral_L | SupraMarginal_L | -3.2961 | 0.0010 |
|  |  | Precentral_L | Rolandic_Oper_R | -3.3768 | 0.0007 |
|  |  | Temporal_Inf_L | Temporal_Sup_L | -3.3499 | 0.0008 |
|  | CON | Occipital_Mid_L | Temporal_Pole_Sup_L | -3.4306 | 0.0006 |
|  |  | Temporal_Inf_L | Temporal_Pole_Sup_L | -3.4306 | 0.0006 |

**Supplementary file XX. Modularity contribution and proportion of edge within and across subnetworks comparing between high and low encoding states.**

| **Subnetwork** | **Modularity**  **contribution** | | **Difference in proportion of edge (high vs low states)** | | | | | | | |
| --- | --- | --- | --- | --- | --- | --- | --- | --- | --- | --- |
|  |  |  | **Type of edge** | | | | **Within vs. Across** | | **ANOVA** | |
|  |  |  | **Within** | | **Across** | |  |  | **state-by-type of edges** | |
|  | *z* | *P* | *z* | *P* | *z* | *P* | *z* | *P* | *F* | *P* |
| SMN | -1.1705 | 0.2418 | -2.4082 | 0.0160 | -1.9508 | 0.0511 | -2.4351 | 0.0149 | 7.0226 | 0.0140 |
| CON | 1.6548 | 0.0980 | 1.5741 | 0.1155 | 0.6592 | 0.5098 | 1.6279 | 0.1036 | 2.6322 | 0.1178 |
| AUD | 0.8745 | 0.3819 | -2.1660 | 0.0303 | -2.3005 | 0.0214 | -1.8162 | 0.0693 | 3.7676 | 0.0641 |
| DMN | 2.0584 | 0.0396 | 1.8431 | 0.0653 | -0.0673 | 0.9464 | 1.8969 | 0.0578 | 3.9919 | 0.0572 |
| VIN | 0.2825 | 0.7775 | -0.2287 | 0.8191 | 0.2556 | 0.7982 | -0.2287 | 0.8191 | 0.0460 | 0.8320 |
| FPN | 1.3050 | 0.1919 | 1.5202 | 0.1285 | 1.2243 | 0.2209 | 1.3588 | 0.1742 | 2.1608 | 0.1546 |
| SAN | -0.8207 | 0.4118 | 0.9552 | 0.3395 | 1.9777 | 0.0480 | 0.4440 | 0.6571 | 0.1098 | 0.7432 |
| SUB | 2.7311 | 0.0063 | 3.0003 | 0.0027 | 2.7580 | 0.0058 | 2.5965 | 0.0094 | 8.4671 | 0.0077 |
| VAN | 0.4440 | 0.6571 | -2.5965 | 0.0094 | -2.6234 | 0.0087 | -2.3005 | 0.0214 | 5.8716 | 0.0233 |
| DAN | 2.3274 | 0.0199 | 0.1480 | 0.8823 | -1.4933 | 0.1353 | 0.9014 | 0.3674 | 0.3392 | 0.5657 |

**Supplementary file 1F. Modularity contribution and proportion of edges within and across subnetworks comparing between high and low encoding states.**

| **Subnetwork** | **Modularity**  **contribution** | | **Difference in proportion of edges (high vs low states)** | | | | | | | |
| --- | --- | --- | --- | --- | --- | --- | --- | --- | --- | --- |
|  |  |  | **Type of edge** | | | | **Within vs. Across** | | **ANOVA** | |
|  |  |  | **Within** | | **Across** | |  |  | **state-by-type of edges** | |
|  | *z* | *P* | *z* | *P* | *z* | *P* | *z* | *P* | *F* | *P* |
| SMN | -1.1705 | 0.2418 | -2.4082 | 0.0160 | -1.9508 | 0.0511 | -2.4351 | 0.0149 | 7.0226 | 0.0140 |
| CON | 1.6548 | 0.0980 | 1.5741 | 0.1155 | 0.6592 | 0.5098 | 1.6279 | 0.1036 | 2.6322 | 0.1178 |
| AUD | 0.8745 | 0.3819 | -2.1660 | 0.0303 | -2.3005 | 0.0214 | -1.8162 | 0.0693 | 3.7676 | 0.0641 |
| DMN | 2.0584 | 0.0396 | 1.8431 | 0.0653 | -0.0673 | 0.9464 | 1.8969 | 0.0578 | 3.9919 | 0.0572 |
| VIN | 0.2825 | 0.7775 | -0.2287 | 0.8191 | 0.2556 | 0.7982 | -0.2287 | 0.8191 | 0.0460 | 0.8320 |
| FPN | 1.3050 | 0.1919 | 1.5202 | 0.1285 | 1.2243 | 0.2209 | 1.3588 | 0.1742 | 2.1608 | 0.1546 |
| SAN | -0.8207 | 0.4118 | 0.9552 | 0.3395 | 1.9777 | 0.0480 | 0.4440 | 0.6571 | 0.1098 | 0.7432 |
| SUB | 2.7311 | 0.0063 | 3.0003 | 0.0027 | 2.7580 | 0.0058 | 2.5965 | 0.0094 | 8.4671 | 0.0077 |
| VAN | 0.4440 | 0.6571 | -2.5965 | 0.0094 | -2.6234 | 0.0087 | -2.3005 | 0.0214 | 5.8716 | 0.0233 |
| DAN | 2.3274 | 0.0199 | 0.1480 | 0.8823 | -1.4933 | 0.1353 | 0.9014 | 0.3674 | 0.3392 | 0.5657 |

**Supplementary file 1G. Five submodules of the DMN**

| **ROI ID** | **Coordinate** | | | **ROI name** | **Submodule ID** |
| --- | --- | --- | --- | --- | --- |
|  | **x** | **y** | **z** |  |  |
| 1 | -41 | -75 | 26 | Occipital_Mid_L | 1 |
| 2 | -13 | -40 | 1 | Precuneus_L | 1 |
| 3 | -46 | -61 | 21 | Temporal_Mid_L | 1 |
| 4 | 43 | -72 | 28 | Occipital_Mid_R | 1 |
| 5 | -7 | -55 | 27 | Precuneus_L | 1 |
| 6 | 6 | -59 | 35 | Precuneus_R | 1 |
| 7 | -11 | -56 | 16 | Precuneus_L | 1 |
| 8 | -3 | -49 | 13 | Precuneus_L | 1 |
| 9 | 8 | -48 | 31 | Cingulum_Mid_R | 1 |
| 10 | 15 | -63 | 26 | Precuneus_R | 1 |
| 11 | -2 | -37 | 44 | Cingulum_Mid_L | 1 |
| 12 | 11 | -54 | 17 | Precuneus_R | 1 |
| 13 | -26 | -40 | -8 | ParaHippocampal_L | 1 |
| 14 | 27 | -37 | -13 | Fusiform_R | 1 |
| 15 | -34 | -38 | -16 | Fusiform_L | 1 |
| 16 | -68 | -23 | -16 | Temporal_Mid_L | 2 |
| 17 | -44 | -65 | 35 | Angular_L | 2 |
| 18 | -39 | -75 | 44 | Angular_L | 2 |
| 19 | -10 | 39 | 52 | Frontal_Sup_Medial_L | 2 |
| 20 | -16 | 29 | 53 | Frontal_Sup_L | 2 |
| 21 | -35 | 20 | 51 | Frontal_Mid_L | 2 |
| 22 | 13 | 55 | 38 | Frontal_Sup_R | 2 |
| 23 | -10 | 55 | 39 | Frontal_Sup_L | 2 |
| 24 | -20 | 45 | 39 | Frontal_Sup_L | 2 |
| 25 | -2 | 38 | 36 | Frontal_Sup_Medial_L | 2 |
| 26 | -20 | 64 | 19 | Frontal_Sup_L | 2 |
| 27 | -58 | -30 | -4 | Temporal_Mid_L | 2 |
| 28 | -68 | -41 | -5 | Temporal_Mid_L | 2 |
| 29 | 13 | 30 | 59 | Frontal_Sup_Medial_R | 2 |
| 30 | 28 | -77 | -32 | Cerebelum_Crus1_R | 2 |
| 31 | -46 | 31 | -13 | Frontal_Inf_Orb_L | 2 |
| 32 | 49 | 35 | -12 | Frontal_Inf_Orb_R | 2 |
| 33 | 8 | 48 | -15 | Rectus_R | 3 |
| 34 | 6 | 54 | 16 | Frontal_Sup_Medial_R | 3 |
| 35 | 6 | 64 | 22 | Frontal_Sup_Medial_R | 3 |
| 36 | -7 | 51 | -1 | Cingulum_Ant_L | 3 |
| 37 | 9 | 54 | 3 | Frontal_Sup_Medial_R | 3 |
| 38 | -3 | 44 | -9 | Frontal_Med_Orb_L | 3 |
| 39 | 8 | 42 | -5 | Frontal_Med_Orb_R | 3 |
| 40 | -11 | 45 | 8 | Cingulum_Ant_L | 3 |
| 41 | -3 | 42 | 16 | Cingulum_Ant_L | 3 |
| 42 | -8 | 48 | 23 | Frontal_Sup_Medial_L | 3 |
| 43 | 12 | 36 | 20 | Cingulum_Ant_R | 3 |
| 44 | -44 | 12 | -34 | Temporal_Pole_Mid_L | 4 |
| 45 | 46 | 16 | -30 | Temporal_Pole_Mid_R | 4 |
| 46 | -56 | -13 | -10 | Temporal_Mid_L | 4 |
| 47 | 52 | -2 | -16 | Temporal_Mid_R | 4 |
| 48 | 52 | 7 | -30 | Temporal_Pole_Mid_R | 4 |
| 49 | -53 | 3 | -27 | Temporal_Mid_L | 4 |
| 50 | -49 | -42 | 1 | Temporal_Mid_L | 4 |
| 51 | 52 | -59 | 36 | Angular_R | 5 |
| 52 | 23 | 33 | 48 | Frontal_Sup_R | 5 |
| 53 | 22 | 39 | 39 | Frontal_Sup_R | 5 |
| 54 | 65 | -12 | -19 | Temporal_Mid_R | 5 |
| 55 | 65 | -31 | -9 | Temporal_Mid_R | 5 |
| 56 | 47 | -50 | 29 | Angular_R | 5 |

**Supplementary file 1H. Robustness of** **graph-analysis results in different window sizes and proportional thresholds.** We confirmed that the results of graph analysis comparing the high and low encoding states were robust across a range of window sizes (i.e., duration of time window; 7.2 s, 14.4 s, 21.6 s, 30 s, 36 s, 45 s, and 60 s) and proportional thresholds (0.1, 0.15, 0.2, and 0.25).

| **Duration of time window** | **Threshold** | ***E_g_*** | | ***E_loc_*** | | **PC** | | **Modularity** | |
| --- | --- | --- | --- | --- | --- | --- | --- | --- | --- |
|  |  | *z* | *P* | *z* | *P* | *z* | *P* | *z* | *P* |
| 10TR (7.2 s) | 0.10 | 3.0270 | 0.0025 | 0.0404 | 0.9678 | 1.8969 | 0.0578 | 1.2512 | 0.2109 |
|  | 0.15 | 2.9732 | 0.0029 | -0.6861 | 0.4926 | 1.0897 | 0.2758 | 0.9552 | 0.3395 |
|  | 0.20 | 3.0808 | 0.0021 | -1.7355 | 0.0827 | 0.8207 | 0.4118 | 1.1705 | 0.2418 |
|  | 0.25 | 3.2154 | 0.0013 | -2.5158 | 0.0119 | 0.8745 | 0.3819 | 1.0090 | 0.3130 |
| 20TR (14.4 s) | 0.10 | 3.2961 | 0.0010 | 1.7624 | 0.0780 | 2.0853 | 0.0370 | 3.1347 | 0.0017 |
|  | 0.15 | 3.3768 | 0.0007 | 0.1480 | 0.8824 | 1.4933 | 0.1353 | 2.6772 | 0.0074 |
|  | 0.20 | 3.5383 | 0.0004 | -1.6010 | 0.1094 | 1.6010 | 0.1094 | 2.6772 | 0.0074 |
|  | 0.25 | 3.5652 | 0.0004 | -3.0808 | 0.0021 | 0.1211 | 0.9036 | 2.5158 | 0.0119 |
| 30TR (21.6 s) | 0.10 | 3.4037 | 0.0007 | 0.6054 | 0.5449 | 2.3813 | 0.0173 | 2.7580 | 0.0058 |
|  | 0.15 | 3.4306 | 0.0006 | -0.5516 | 0.5812 | 2.4082 | 0.0160 | 2.2467 | 0.0247 |
|  | 0.20 | 3.4844 | 0.0005 | -2.3813 | 0.0173 | 3.0539 | 0.0023 | 2.0584 | 0.0396 |
|  | 0.25 | 3.4306 | 0.0006 | -3.0270 | 0.0025 | 1.7624 | 0.0780 | 1.9508 | 0.0511 |
| 41TR (30 s) | 0.10 | 4.1571 | 0.0001 | 0.1480 | 0.8824 | 3.0808 | 0.0021 | 2.5427 | 0.0110 |
|  | 0.15 | 4.2917 | 0.0001 | -1.3588 | 0.1742 | 2.8118 | 0.0049 | 2.3005 | 0.0214 |
|  | 0.20 | 4.2917 | 0.0001 | -2.8118 | 0.0049 | 1.6817 | 0.0926 | 2.1122 | 0.0347 |
|  | 0.25 | 4.2647 | 0.0001 | -4.0495 | 0.0001 | 2.1660 | 0.0303 | 1.8700 | 0.0615 |
| 50TR (36 s) | 0.10 | 3.7266 | 0.0002 | 1.4933 | 0.1353 | 2.8387 | 0.0045 | 3.1077 | 0.0019 |
|  | 0.15 | 3.9688 | 0.0001 | -0.6861 | 0.4926 | 2.7580 | 0.0058 | 2.5427 | 0.0110 |
|  | 0.20 | 3.9688 | 0.0001 | -2.4620 | 0.0138 | 2.7041 | 0.0068 | 2.3274 | 0.0199 |
|  | 0.25 | 4.1840 | 0.0001 | -3.8073 | 0.0001 | 2.1391 | 0.0324 | 2.0584 | 0.0396 |
| 62TR (45 s) | 0.10 | 4.2109 | 0.0001 | 1.7086 | 0.0875 | 1.9508 | 0.0511 | 3.2423 | 0.0012 |
|  | 0.15 | 4.2378 | 0.0001 | -0.1211 | 0.9036 | 1.7086 | 0.0875 | 3.0539 | 0.0023 |
|  | 0.20 | 4.2647 | 0.0001 | -2.8387 | 0.0045 | 1.9777 | 0.0480 | 2.8387 | 0.0045 |
|  | 0.25 | 4.2647 | 0.0001 | -4.0226 | 0.0001 | 1.8162 | 0.0693 | 2.3274 | 0.0199 |
| 83TR (60 s) | 0.10 | 3.1347 | 0.0017 | 0.1749 | 0.8612 | 1.4126 | 0.1578 | 3.2692 | 0.0011 |
|  | 0.15 | 3.6190 | 0.0003 | -1.1974 | 0.2312 | 1.1435 | 0.2528 | 2.7849 | 0.0054 |
|  | 0.20 | 3.7266 | 0.0002 | -2.1660 | 0.0303 | 1.4933 | 0.1353 | 2.6234 | 0.0087 |
|  | 0.25 | 3.5921 | 0.0003 | -3.4306 | 0.0006 | 1.4395 | 0.1500 | 2.1929 | 0.0283 |

*Notes*. Statistics are derived from Wilcoxon signed-rank tests. Graph metrics (*E_g_*: global efficiency; *E_loc_*: local efficiency; PC: participation coefficient; Modularity) were computed at the entire-network level.

**Supplementary file 1I.** **Results of graph analysis using overlapping sliding windows.** We confirmed that the results of graph analysis comparing the high and low encoding states were unchanged when we used sliding windows (in steps of 1 TR, resulting in 2,100 windows per participant).

| **Duration of time window** | **Threshold** | ***E_g_*** | | ***E_loc_*** | | **PC** | | **Modularity** | |
| --- | --- | --- | --- | --- | --- | --- | --- | --- | --- |
|  |  | *z* | *P* | *z* | *P* | *z* | *P* | *z* | *P* |
| 36 s | 0.10 | 3.7266 | 0.0002 | 1.4933 | 0.1353 | 2.1391 | 0.0324 | 3.1885 | 0.0014 |
|  | 0.15 | 3.9688 | 0.0001 | -0.6861 | 0.4926 | 2.2467 | 0.0247 | 2.4620 | 0.0138 |
|  | 0.20 | 3.9688 | 0.0001 | -2.4620 | 0.0138 | 3.4037 | 0.0007 | 2.4351 | 0.0149 |
|  | 0.25 | 4.1840 | 0.0001 | -3.8073 | 0.0001 | 2.6234 | 0.0087 | 1.8700 | 0.0615 |

*Notes*. Statistics are derived from Wilcoxon signed-rank tests. Graph metrics (*E_g_*: global efficiency; *E_loc_*: local efficiency; PC: participation coefficient; Modularity) were computed at the entire-network level.

**Supplementary file 1J. Results of graph analysis using time series shifted by 5 s.** We confirmed that the results of graph analysis comparing the high and low encoding states were unchanged when we shifted time series (before dividing it into short time windows) by 5 s to take the hemodynamic delay of BOLD response into account (5s, according to canonical hemodynamic response function provided by SPM12).

| **Duration of time window** | **Threshold** | ***E_g_*** | | ***E_loc_*** | | **PC** | | **Modularity** | |
| --- | --- | --- | --- | --- | --- | --- | --- | --- | --- |
|  |  | *z* | *P* | *z* | *P* | *z* | *P* | *z* | *P* |
| 36 s | 0.10 | 3.7266 | 0.0002 | 1.4933 | 0.1353 | 2.5427 | 0.0110 | 3.0270 | 0.0025 |
|  | 0.15 | 3.9688 | 0.0001 | -0.6861 | 0.4926 | 2.3544 | 0.0186 | 2.6503 | 0.0080 |
|  | 0.20 | 3.9688 | 0.0001 | -2.4620 | 0.0138 | 2.2467 | 0.0247 | 2.3274 | 0.0199 |
|  | 0.25 | 4.1840 | 0.0001 | -3.8073 | 0.0001 | 2.7041 | 0.0068 | 1.7893 | 0.0736 |

*Notes*. Statistics are derived from Wilcoxon signed-rank tests. Graph metrics (*E_g_*: global efficiency; *E_loc_*: local efficiency; PC: participation coefficient; Modularity) were computed at the entire-network level.

**Supplementary file 1K. Results of graph analysis using time series that contain task-related signals.** We confirmed that the results of graph analysis comparing the high and low encoding states were unchanged when we used time series that kept task-related signals instead of residual time series.

| **Duration of time window** | **Threshold** | ***E_g_*** | | ***E_loc_*** | | **PC** | | **Modularity** | |
| --- | --- | --- | --- | --- | --- | --- | --- | --- | --- |
|  |  | *z* | *P* | *z* | *P* | *z* | *P* | *z* | *P* |
| 36 s | 0.10 | 3.6728 | 0.0002 | 1.5471 | 0.1218 | 1.4395 | 0.1500 | 3.0539 | 0.0023 |
|  | 0.15 | 3.9957 | 0.0001 | -0.6861 | 0.4926 | 2.6772 | 0.0074 | 2.5696 | 0.0102 |
|  | 0.20 | 3.9688 | 0.0001 | -2.4889 | 0.0128 | 2.2736 | 0.0230 | 2.3813 | 0.0173 |
|  | 0.25 | 4.1571 | 0.0001 | -3.8073 | 0.0001 | 2.0853 | 0.0370 | 1.8431 | 0.0653 |

*Notes*. Statistics are derived from Wilcoxon signed-rank tests. Graph metrics (*E_g_*: global efficiency; *E_loc_*: local efficiency; PC: participation coefficient; Modularity) were computed at the entire-network level.

**Supplementary file 1L. Results of graph analysis including low-confidence hit trials.** We confirmed that the results of graph analysis comparing the high and low encoding states were unchanged when we defined the window-wise encoding performance by using both high- and low-confidence hit trials (i.e., the number of HH plus LH trials divided by that of the picture trials, computed within each time window).

| **Duration of time window** | **Threshold** | ***E_g_*** | | ***E_loc_*** | | **PC** | | **Modularity** | |
| --- | --- | --- | --- | --- | --- | --- | --- | --- | --- |
|  |  | *z* | *P* | *z* | *P* | *z* | *P* | *z* | *P* |
| 36 s | 0.10 | 3.1077 | 0.0019 | 2.1660 | 0.0303 | 0.7938 | 0.4273 | 2.7311 | 0.0063 |
|  | 0.15 | 3.0270 | 0.0025 | 0.9014 | 0.3674 | 1.1166 | 0.2641 | 2.0853 | 0.0370 |
|  | 0.20 | 3.0539 | 0.0023 | -0.7668 | 0.4432 | 0.2556 | 0.7982 | 1.6817 | 0.0926 |
|  | 0.25 | 3.1347 | 0.0017 | -2.2736 | 0.0230 | 0.9821 | 0.3260 | 1.0628 | 0.2879 |

*Notes*. Statistics are derived from Wilcoxon signed-rank tests. Graph metrics (*E_g_*: global efficiency; *E_loc_*: local efficiency; PC: participation coefficient; Modularity) were computed at the entire-network level.

**Supplementary file 1M. Graph-analysis results using tertiles to classify high and low encoding states.** In this control analysis, we classified the time windows based on participant-specific tertiles of window-wise encoding performance, and compared graph metrics between the top and bottom tertiles. The proportion of HH trials was 69.7% ± 16.3% (5.58 ± 1.33 trials per window) for the top tertile and 28.4 ± 14.4% (2.24 ± 1.13 trials per window) for the bottom tertile.

| **Duration of time window** | **Threshold** | ***E_g_*** | | ***E_loc_*** | | **PC** | | **Modularity** | |
| --- | --- | --- | --- | --- | --- | --- | --- | --- | --- |
|  |  | *z* | *P* | *z* | *P* | *z* | *P* | *z* | *P* |
| 41TR (30 s) | 0.10 | 4.2647 | 0.0001 | 0.8207 | 0.4118 | 2.3274 | 0.0199 | 2.3005 | 0.0214 |
|  | 0.15 | 4.2647 | 0.0001 | -1.2243 | 0.2209 | 2.1929 | 0.0283 | 2.2467 | 0.0247 |
|  | 0.20 | 4.2647 | 0.0001 | -3.5383 | 0.0004 | 2.6772 | 0.0074 | 1.8969 | 0.0578 |
|  | 0.25 | 4.2647 | 0.0001 | -4.0764 | 0.0000 | 2.3813 | 0.0173 | 1.6279 | 0.1036 |
| 50TR (36 s) | 0.10 | 3.7804 | 0.0002 | 1.6817 | 0.0926 | 2.6234 | 0.0087 | 3.6997 | 0.0002 |
|  | 0.15 | 3.8611 | 0.0001 | -0.9821 | 0.3260 | 2.9732 | 0.0029 | 3.0808 | 0.0021 |
|  | 0.20 | 3.8342 | 0.0001 | -2.8656 | 0.0042 | 2.7041 | 0.0068 | 2.9194 | 0.0035 |
|  | 0.25 | 3.9419 | 0.0001 | -3.4575 | 0.0005 | 3.4306 | 0.0006 | 2.1929 | 0.0283 |
| 62TR (45 s) | 0.10 | 3.6997 | 0.0002 | 1.1974 | 0.2312 | 2.1929 | 0.0283 | 3.0808 | 0.0021 |
|  | 0.15 | 3.7804 | 0.0002 | -1.1166 | 0.2641 | 2.5696 | 0.0102 | 2.7311 | 0.0063 |
|  | 0.20 | 3.8611 | 0.0001 | -3.5652 | 0.0004 | 2.2467 | 0.0247 | 2.5427 | 0.0110 |
|  | 0.25 | 3.9688 | 0.0001 | -3.8880 | 0.0001 | 2.8656 | 0.0042 | 2.1929 | 0.0283 |
| 83TR (60 s) | 0.10 | 3.1077 | 0.0019 | 0.7668 | 0.4432 | 2.0853 | 0.0370 | 2.8925 | 0.0038 |
|  | 0.15 | 3.4037 | 0.0007 | -1.1705 | 0.2418 | 1.4126 | 0.1578 | 2.4620 | 0.0138 |
|  | 0.20 | 3.3230 | 0.0009 | -2.5696 | 0.0102 | 1.3319 | 0.1829 | 2.1660 | 0.0303 |
|  | 0.25 | 3.3499 | 0.0008 | -3.1885 | 0.0014 | 1.8431 | 0.0653 | 1.9238 | 0.0544 |

*Notes*. Statistics are derived from Wilcoxon signed-rank tests. Graph metrics (*E_g_*: global efficiency; *E_loc_*: local efficiency; PC: participation coefficient; Modularity) were computed at the entire-network level.

**Supplementary file 1N. Graph-analysis results using quartiles to classify high and low encoding states.** In this control analysis, we classified the time windows based on participant-specific quartiles of window-wise encoding performance, and compared graph metrics between the top and bottom quartiles. The proportion of HH trials was 73.2% ± 16.2% (5.91 ± 1.37 trials per window) for the top quartile and 24.7 ± 14.1% (1.94 ± 1.11 trials per window) for the bottom quartile.

| **Duration of time window** | **Threshold** | ***E_g_*** | | ***E_loc_*** | | **PC** | | **Modularity** | |
| --- | --- | --- | --- | --- | --- | --- | --- | --- | --- |
|  |  | *z* | *P* | *z* | *P* | *z* | *P* | *z* | *P* |
| 41TR (30 s) | 0.10 | 4.3186 | 0.0001 | -0.2287 | 0.8191 | 3.0539 | 0.0023 | 1.8700 | 0.0615 |
|  | 0.15 | 4.3186 | 0.0001 | -1.7624 | 0.0780 | 2.3274 | 0.0199 | 1.6279 | 0.1036 |
|  | 0.20 | 4.2917 | 0.0001 | -3.6997 | 0.0002 | 2.6234 | 0.0087 | 1.3588 | 0.1742 |
|  | 0.25 | 4.2917 | 0.0001 | -4.1571 | 0.0000 | 1.8700 | 0.0615 | 0.8476 | 0.3967 |
| 50TR (36 s) | 0.10 | 3.4306 | 0.0006 | 1.0090 | 0.3130 | 2.1122 | 0.0347 | 3.3768 | 0.0007 |
|  | 0.15 | 3.6190 | 0.0003 | -0.8745 | 0.3819 | 2.6234 | 0.0087 | 2.7580 | 0.0058 |
|  | 0.20 | 3.5114 | 0.0004 | -2.8387 | 0.0045 | 2.5158 | 0.0119 | 2.2198 | 0.0264 |
|  | 0.25 | 3.6190 | 0.0003 | -3.3230 | 0.0009 | 2.5158 | 0.0119 | 1.6817 | 0.0926 |
| 62TR (45 s) | 0.10 | 3.6997 | 0.0002 | 1.2781 | 0.2012 | 1.6279 | 0.1036 | 3.2154 | 0.0013 |
|  | 0.15 | 3.8073 | 0.0001 | -0.9552 | 0.3395 | 3.0270 | 0.0025 | 3.1347 | 0.0017 |
|  | 0.20 | 3.8880 | 0.0001 | -3.3768 | 0.0007 | 3.1077 | 0.0019 | 2.9194 | 0.0035 |
|  | 0.25 | 4.0226 | 0.0001 | -3.7266 | 0.0002 | 3.1347 | 0.0017 | 2.6234 | 0.0087 |
| 83TR (60 s) | 0.10 | 3.4844 | 0.0005 | 0.2825 | 0.7775 | 1.7355 | 0.0827 | 3.0808 | 0.0021 |
|  | 0.15 | 3.5383 | 0.0004 | -1.4395 | 0.1500 | 1.5471 | 0.1218 | 2.7041 | 0.0068 |
|  | 0.20 | 3.6190 | 0.0003 | -3.1347 | 0.0017 | 1.7086 | 0.0875 | 2.5696 | 0.0102 |
|  | 0.25 | 3.5652 | 0.0004 | -3.6997 | 0.0002 | 2.1122 | 0.0347 | 2.0315 | 0.0422 |

*Notes*. Statistics are derived from Wilcoxon signed-rank tests. Graph metrics (*E_g_*: global efficiency; *E_loc_*: local efficiency; PC: participation coefficient; Modularity) were computed at the entire-network level.**Supplementary file 1O. Results of graph analysis controlling for time passed within a session or across sessions.** We confirmed that the results of graph analysis comparing the high and low encoding states were unchanged when we regressed out the effect of the amount of time passed within sessions (defined by a dummy variable denoting the order of windows [1, 2, 3, … 15] in each session) or time passed across sessions (defined by a dummy variable denoting session ID [1, 1, … 2, 2, … 3, 3, …]) on a window-by-window basis.

| **Covariate** | ***E_g_*** | | ***E_loc_*** | | **PC** | | **Modularity** | |
| --- | --- | --- | --- | --- | --- | --- | --- | --- |
|  | *z* | *P* | *z* | *P* | *z* | *P* | *z* | *P* |
| Time passed within each session, linear | 3.4575 | 0.0005 | 0.3094 | 0.7570 | 2.0853 | 0.0370 | 3.4575 | 0.0005 |
| Time passed within each session, quadratic | 3.9150 | 0.0001 | -0.7130 | 0.4758 | 2.8387 | 0.0045 | 3.9150 | 0.0001 |
| Time passed across sessions, linear | 3.9957 | 0.0001 | -0.7938 | 0.4273 | 2.5427 | 0.0110 | 3.9957 | 0.0001 |
| Time passed across sessions, quadratic | 3.9957 | 0.0001 | -1.0359 | 0.3002 | 2.7041 | 0.0068 | 3.9957 | 0.0001 |

*Notes*. Statistics are derived from Wilcoxon signed-rank tests. Graph metrics (*E_g_*: global efficiency; *E_loc_*: local efficiency; PC: participation coefficient; Modularity) were computed at the entire-network level.

**Supplementary file 1P. Results of control analysis testing effect of proportion of picture trials.** In this control analysis, we classified the time windows based on the proportion of the picture trials and compared the graph metrics between “more pic” and “fewer pic” periods.

| **Duration of time window** | **Threshold** | ***E_g_*** | | ***E_loc_*** | | **PC** | | **Modularity** | |
| --- | --- | --- | --- | --- | --- | --- | --- | --- | --- |
|  |  | *z* | *P* | *z* | *P* | *z* | *P* | *z* | *P* |
| 36 s | 0.10 | 0.9552 | 0.3395 | 0.2825 | 0.7775 | -0.3902 | 0.6964 | 1.2781 | 0.2012 |
|  | 0.15 | 0.8207 | 0.4118 | 0.2287 | 0.8191 | -0.2825 | 0.7775 | 1.3319 | 0.1829 |
|  | 0.20 | 0.5785 | 0.5629 | -0.5516 | 0.5812 | 0.2287 | 0.8191 | 1.3050 | 0.1919 |
|  | 0.25 | 0.6592 | 0.5098 | -0.4709 | 0.6377 | -0.0673 | 0.9464 | 1.0090 | 0.3130 |

*Notes*. Statistics are derived from Wilcoxon signed-rank tests. Graph metrics (*E_g_*: global efficiency; *E_loc_*: local efficiency; PC: participation coefficient; Modularity) were computed at the entire-network level.

**Supplementary file 1Q. Results of control analysis testing effect of reaction time for semantic judgment.** In this control analysis, we classified the time windows based on mean RT (computed within each time window) for semantic judgment and compared the graph metrics between “longer RT” and “shorter RT” periods.

| **Duration of time window** | **Threshold** | ***E_g_*** | | ***E_loc_*** | | **PC** | | **Modularity** | |
| --- | --- | --- | --- | --- | --- | --- | --- | --- | --- |
|  |  | *z* | *P* | *z* | *P* | *z* | *P* | *z* | *P* |
| 36 s | 0.10 | -0.4978 | 0.6186 | 0.5247 | 0.5998 | -0.1749 | 0.8612 | -1.4395 | 0.1500 |
|  | 0.15 | -0.7130 | 0.4758 | 0.2556 | 0.7982 | -0.0404 | 0.9678 | -1.0090 | 0.3130 |
|  | 0.20 | -0.7399 | 0.4593 | 1.5741 | 0.1155 | -0.4440 | 0.6571 | -1.1166 | 0.2641 |
|  | 0.25 | -0.6323 | 0.5272 | 1.1166 | 0.2641 | -0.6861 | 0.4926 | -0.7130 | 0.4758 |

*Notes*. Statistics are derived from Wilcoxon signed-rank tests. Graph metrics (*E_g_*: global efficiency; *E_loc_*: local efficiency; PC: participation coefficient; Modularity) were computed at the entire-network level.

**Supplementary file 1R. 285 ROIs included in 11 subnetworks of the Gordon atlas.**

| **ROI ID** | **MNI coordinates** | | | **Assigned network** |
| --- | --- | --- | --- | --- |
|  | ***x*** | ***y*** | ***z*** |  |
| 1 | -18.80 | -48.70 | 65.00 | SMN |
| 2 | -10.70 | -47.50 | 60.30 | SMN |
| 3 | -15.60 | -33.10 | 66.10 | SMN |
| 4 | -10.90 | -29.30 | 69.50 | SMN |
| 5 | -6.60 | -20.40 | 74.20 | SMN |
| 6 | -10.80 | -41.10 | 64.90 | SMN |
| 7 | -5.00 | -28.20 | 60.40 | SMN |
| 8 | -5.40 | -15.90 | 48.80 | SMN |
| 9 | -35.80 | -29.70 | 54.50 | SMN |
| 10 | -36.80 | -22.80 | 61.90 | SMN |
| 11 | -20.50 | -24.90 | 64.50 | SMN |
| 12 | -23.40 | -13.80 | 64.20 | SMN |
| 13 | -17.20 | -8.60 | 67.90 | SMN |
| 14 | -28.60 | -44.70 | 61.70 | SMN |
| 15 | -54.10 | -21.30 | 40.80 | SMN |
| 16 | -35.20 | -35.30 | 42.00 | SMN |
| 17 | -27.50 | -37.20 | 61.40 | SMN |
| 18 | -47.20 | -31.40 | 54.80 | SMN |
| 19 | 20.80 | -48.20 | 66.10 | SMN |
| 20 | 16.50 | -32.80 | 67.70 | SMN |
| 21 | 4.80 | -27.10 | 64.80 | SMN |
| 22 | 11.90 | -40.70 | 67.00 | SMN |
| 23 | 5.10 | -17.10 | 51.60 | SMN |
| 24 | 6.80 | -8.10 | 50.90 | SMN |
| 25 | 38.10 | -22.40 | 60.30 | SMN |
| 26 | 19.70 | -25.00 | 65.20 | SMN |
| 27 | 12.40 | -28.30 | 69.60 | SMN |
| 28 | 29.20 | -13.50 | 64.20 | SMN |
| 29 | 17.00 | -16.90 | 70.90 | SMN |
| 30 | 20.90 | -6.40 | 65.00 | SMN |
| 31 | 29.50 | -42.50 | 60.40 | SMN |
| 32 | 34.20 | -40.60 | 51.60 | SMN |
| 33 | 39.60 | -31.50 | 39.70 | SMN |
| 34 | 28.00 | -34.80 | 63.10 | SMN |
| 35 | 39.20 | -34.60 | 57.50 | SMN |
| 36 | 37.30 | -25.90 | 50.90 | SMN |
| 37 | 48.70 | -26.10 | 52.20 | SMN |
| 38 | 53.00 | -22.70 | 39.10 | SMN |
| 39 | -51.80 | -7.80 | 38.50 | SMN |
| 40 | -41.50 | -12.50 | 50.40 | SMN |
| 41 | -51.50 | -11.90 | 29.70 | SMN |
| 42 | -46.10 | -17.80 | 52.70 | SMN |
| 43 | 49.60 | -7.40 | 36.10 | SMN |
| 44 | 42.30 | -11.00 | 47.30 | SMN |
| 45 | 53.90 | -8.30 | 26.10 | SMN |
| 46 | 47.80 | -15.10 | 49.30 | SMN |
| 47 | -16.60 | -36.10 | 42.70 | CON |
| 48 | -9.40 | -0.10 | 42.90 | CON |
| 49 | -8.40 | 14.60 | 33.80 | CON |
| 50 | -9.00 | 25.30 | 27.70 | CON |
| 51 | -8.00 | -8.70 | 62.90 | CON |
| 52 | -42.10 | -4.50 | 47.30 | CON |
| 53 | -57.70 | -40.60 | 35.80 | CON |
| 54 | -38.70 | -16.00 | -5.30 | CON |
| 55 | -39.10 | -1.60 | -12.20 | CON |
| 56 | -37.70 | 2.90 | 11.70 | CON |
| 57 | -36.60 | 1.40 | 6.40 | CON |
| 58 | -37.30 | 8.90 | -0.90 | CON |
| 59 | -28.80 | 23.70 | 8.40 | CON |
| 60 | -59.80 | -4.10 | 8.80 | CON |
| 61 | -55.10 | -32.30 | 23.00 | CON |
| 62 | -58.80 | -23.90 | 31.00 | CON |
| 63 | -51.80 | -0.60 | 5.00 | CON |
| 64 | -48.60 | 7.50 | 11.10 | CON |
| 65 | -26.60 | 46.80 | 20.90 | CON |
| 66 | -28.80 | 38.30 | 28.20 | CON |
| 67 | 16.20 | -33.10 | 43.20 | CON |
| 68 | 6.70 | 5.00 | 55.90 | CON |
| 69 | 8.60 | 4.20 | 40.10 | CON |
| 70 | 8.80 | 10.80 | 45.90 | CON |
| 71 | 6.00 | 21.80 | 32.40 | CON |
| 72 | 16.20 | 0.80 | 67.50 | CON |
| 73 | 8.00 | -6.20 | 63.70 | CON |
| 74 | 42.50 | -2.30 | 47.20 | CON |
| 75 | 57.50 | -40.30 | 34.70 | CON |
| 76 | 54.90 | -27.00 | 29.60 | CON |
| 77 | 38.80 | -14.40 | -5.00 | CON |
| 78 | 39.70 | 1.20 | -13.10 | CON |
| 79 | 36.70 | 5.20 | 12.70 | CON |
| 80 | 39.60 | 10.40 | -1.60 | CON |
| 81 | 36.50 | 5.70 | 6.00 | CON |
| 82 | 33.70 | 22.60 | 3.70 | CON |
| 83 | 34.00 | 24.40 | 10.00 | CON |
| 84 | 50.10 | 3.00 | 3.90 | CON |
| 85 | 24.40 | 50.80 | 24.30 | CON |
| 86 | 31.30 | 39.70 | 25.60 | CON |
| 87 | -6.10 | -26.00 | 28.50 | CON |
| 88 | -12.70 | -64.90 | 31.80 | CON |
| 89 | -10.90 | -73.40 | 42.90 | CON |
| 90 | 7.60 | -27.00 | 28.40 | CON |
| 91 | 15.60 | -69.50 | 39.60 | CON |
| 92 | -32.00 | -29.30 | 15.60 | AUD |
| 93 | -46.30 | -41.40 | 25.90 | AUD |
| 94 | -35.80 | -33.50 | 19.90 | AUD |
| 95 | -52.70 | -20.60 | 5.40 | AUD |
| 96 | -59.60 | -38.50 | 16.50 | AUD |
| 97 | -58.70 | -29.90 | 11.10 | AUD |
| 98 | -40.60 | -38.30 | 14.50 | AUD |
| 99 | -33.70 | -21.80 | 9.90 | AUD |
| 100 | -37.20 | -14.00 | 19.40 | AUD |
| 101 | -52.20 | -14.10 | 15.20 | AUD |
| 102 | -50.60 | -22.40 | 19.20 | AUD |
| 103 | -54.40 | -1.40 | -0.70 | AUD |
| 104 | 33.60 | -22.30 | 13.00 | AUD |
| 105 | 36.40 | -30.70 | 19.40 | AUD |
| 106 | 53.80 | -15.80 | 5.20 | AUD |
| 107 | 59.20 | -38.60 | 14.60 | AUD |
| 108 | 61.70 | -24.00 | 1.30 | AUD |
| 109 | 60.00 | -25.20 | 10.20 | AUD |
| 110 | 38.40 | -12.20 | 20.00 | AUD |
| 111 | 36.60 | -10.00 | 12.40 | AUD |
| 112 | 60.90 | -2.20 | 10.70 | AUD |
| 113 | 54.20 | -13.60 | 16.90 | AUD |
| 114 | 39.70 | -22.50 | 2.60 | AUD |
| 115 | 55.80 | 2.00 | -2.00 | AUD |
| 116 | -11.20 | -52.40 | 36.50 | DMN |
| 117 | -11.70 | 26.70 | 57.00 | DMN |
| 118 | -47.20 | -58.00 | 30.80 | DMN |
| 119 | -5.60 | 42.20 | 35.10 | DMN |
| 120 | -1.70 | -17.70 | 39.10 | DMN |
| 121 | -19.50 | 30.10 | 45.50 | DMN |
| 122 | -39.30 | -73.90 | 38.30 | DMN |
| 123 | -27.50 | 53.60 | 0.00 | DMN |
| 124 | -5.90 | 54.80 | -11.30 | DMN |
| 125 | -6.80 | 38.20 | -9.40 | DMN |
| 126 | -63.20 | -28.70 | -7.20 | DMN |
| 127 | -53.10 | -11.40 | -16.00 | DMN |
| 128 | -15.90 | 48.60 | 37.20 | DMN |
| 129 | -19.50 | 56.30 | 27.50 | DMN |
| 130 | -6.50 | 54.70 | 18.10 | DMN |
| 131 | -15.70 | 64.70 | 13.70 | DMN |
| 132 | -6.00 | 44.90 | 6.30 | DMN |
| 133 | -26.20 | 26.60 | 38.80 | DMN |
| 134 | -29.30 | 16.80 | 50.70 | DMN |
| 135 | -41.70 | 16.10 | 47.50 | DMN |
| 136 | 12.30 | -51.60 | 34.50 | DMN |
| 137 | 11.90 | 21.90 | 59.90 | DMN |
| 138 | 7.70 | 44.10 | 5.50 | DMN |
| 139 | 3.00 | -19.60 | 37.90 | DMN |
| 140 | 21.90 | 21.00 | 46.20 | DMN |
| 141 | 48.90 | -53.00 | 28.60 | DMN |
| 142 | 62.50 | -25.60 | -5.50 | DMN |
| 143 | 7.40 | -69.30 | 49.90 | DMN |
| 144 | 46.50 | -67.30 | 36.20 | DMN |
| 145 | 7.20 | 48.40 | -10.10 | DMN |
| 146 | 57.50 | -7.40 | -16.40 | DMN |
| 147 | 21.00 | 32.80 | 42.10 | DMN |
| 148 | 21.40 | 42.80 | 35.10 | DMN |
| 149 | 16.00 | 61.00 | 19.80 | DMN |
| 150 | 8.20 | 53.80 | 14.00 | DMN |
| 151 | 5.90 | 54.90 | 29.40 | DMN |
| 152 | 13.80 | 46.70 | 42.10 | DMN |
| 153 | 6.80 | 44.50 | 34.80 | DMN |
| 154 | 30.60 | 18.90 | 48.70 | DMN |
| 155 | 54.40 | 1.10 | -12.90 | DMN |
| 156 | -18.40 | -85.50 | 21.60 | VIN |
| 157 | -16.80 | -60.10 | -5.40 | VIN |
| 158 | -11.30 | -83.20 | 3.90 | VIN |
| 159 | -22.00 | -58.10 | 1.50 | VIN |
| 160 | -9.60 | -58.00 | 3.00 | VIN |
| 161 | -16.70 | -46.00 | -3.70 | VIN |
| 162 | -13.70 | -77.40 | 26.60 | VIN |
| 163 | -31.30 | -84.20 | 9.00 | VIN |
| 164 | -34.20 | -86.60 | -0.50 | VIN |
| 165 | -43.40 | -67.60 | 9.70 | VIN |
| 166 | -28.80 | -58.80 | -9.10 | VIN |
| 167 | -34.40 | -63.90 | -15.70 | VIN |
| 168 | -34.30 | -43.80 | -21.60 | VIN |
| 169 | -5.40 | -88.00 | 18.60 | VIN |
| 170 | -8.60 | -77.50 | -3.50 | VIN |
| 171 | -41.20 | -72.10 | -5.90 | VIN |
| 172 | -25.20 | -97.20 | -7.90 | VIN |
| 173 | -22.60 | -81.70 | -11.70 | VIN |
| 174 | 22.00 | -84.60 | 23.70 | VIN |
| 175 | 22.30 | -46.50 | -9.90 | VIN |
| 176 | 15.50 | -74.10 | 9.40 | VIN |
| 177 | 19.60 | -45.30 | -4.40 | VIN |
| 178 | 15.60 | -59.60 | -5.00 | VIN |
| 179 | 26.80 | -55.00 | 54.20 | VIN |
| 180 | 17.60 | -78.30 | 34.00 | VIN |
| 181 | 7.70 | -85.60 | 31.60 | VIN |
| 182 | 35.40 | -77.10 | 21.10 | VIN |
| 183 | 31.70 | -85.70 | 2.40 | VIN |
| 184 | 43.80 | -67.20 | 2.00 | VIN |
| 185 | 47.30 | -52.40 | -11.70 | VIN |
| 186 | 49.00 | -54.50 | 8.80 | VIN |
| 187 | 31.20 | -45.60 | -5.80 | VIN |
| 188 | 26.90 | -69.10 | -6.60 | VIN |
| 189 | 34.90 | -44.00 | -20.00 | VIN |
| 190 | 13.80 | -92.30 | 14.70 | VIN |
| 191 | 10.50 | -73.80 | -1.50 | VIN |
| 192 | 20.40 | -87.30 | -6.60 | VIN |
| 193 | 5.10 | -80.20 | 23.10 | VIN |
| 194 | 14.60 | -70.30 | 23.30 | VIN |
| 195 | -38.10 | 48.80 | 10.50 | FPN |
| 196 | -55.90 | -47.70 | -9.30 | FPN |
| 197 | -5.50 | 29.30 | 44.00 | FPN |
| 198 | -40.30 | 50.40 | -4.80 | FPN |
| 199 | -34.10 | -61.00 | 42.40 | FPN |
| 200 | -43.00 | 19.40 | 33.50 | FPN |
| 201 | -40.20 | 23.60 | 23.30 | FPN |
| 202 | -21.30 | 63.10 | 1.90 | FPN |
| 203 | -28.60 | 50.90 | 10.10 | FPN |
| 204 | 47.90 | -42.50 | 41.50 | FPN |
| 205 | 38.10 | 45.90 | 7.70 | FPN |
| 206 | 59.70 | -41.00 | -10.90 | FPN |
| 207 | 7.00 | 25.70 | 47.30 | FPN |
| 208 | 42.80 | 48.30 | -5.10 | FPN |
| 209 | 41.50 | -53.50 | 44.00 | FPN |
| 210 | 35.70 | -56.70 | 45.20 | FPN |
| 211 | 37.80 | 28.70 | 35.60 | FPN |
| 212 | 41.80 | 29.10 | 21.60 | FPN |
| 213 | 38.60 | 18.80 | 25.50 | FPN |
| 214 | 28.40 | 57.00 | -5.10 | FPN |
| 215 | 23.50 | 59.10 | 4.90 | FPN |
| 216 | 30.90 | 52.20 | 9.90 | FPN |
| 217 | 42.40 | 19.50 | 48.20 | FPN |
| 218 | 38.90 | 9.60 | 42.70 | FPN |
| 219 | -10.00 | 33.90 | 21.50 | SAN |
| 220 | -32.50 | 17.20 | -7.80 | SAN |
| 221 | 8.40 | 34.70 | 22.60 | SAN |
| 222 | 30.60 | 22.80 | -4.70 | SAN |
| 223 | -3.80 | 12.10 | 64.60 | VAT |
| 224 | -44.80 | -54.00 | 14.60 | VAT |
| 225 | -51.60 | -55.90 | 11.40 | VAT |
| 226 | -48.10 | -40.00 | 2.40 | VAT |
| 227 | -50.00 | 20.80 | 10.60 | VAT |
| 228 | -47.20 | 39.00 | -9.10 | VAT |
| 229 | -29.10 | 20.50 | -14.00 | VAT |
| 230 | -44.30 | 33.20 | -7.20 | VAT |
| 231 | -45.40 | 28.80 | 0.80 | VAT |
| 232 | -38.70 | 4.80 | 48.40 | VAT |
| 233 | -59.00 | -18.00 | -3.00 | VAT |
| 234 | 57.50 | -45.30 | 9.00 | VAT |
| 235 | 60.90 | -38.70 | 1.70 | VAT |
| 236 | 57.10 | -17.00 | -2.60 | VAT |
| 237 | 47.40 | -39.60 | 13.20 | VAT |
| 238 | 45.50 | -37.30 | 3.40 | VAT |
| 239 | 48.50 | -26.50 | -0.10 | VAT |
| 240 | 52.50 | 23.70 | 10.30 | VAT |
| 241 | 48.10 | 38.30 | -9.20 | VAT |
| 242 | 45.20 | 30.70 | -5.60 | VAT |
| 243 | 27.40 | 19.70 | -14.90 | VAT |
| 244 | 57.10 | -6.30 | -7.70 | VAT |
| 245 | 46.60 | -21.50 | -8.50 | VAT |
| 246 | -27.30 | -6.80 | 46.30 | DAT |
| 247 | -27.30 | 1.90 | 52.90 | DAT |
| 248 | -19.80 | 6.40 | 55.70 | DAT |
| 249 | -21.30 | -0.20 | 62.70 | DAT |
| 250 | -31.10 | -48.90 | 47.10 | DAT |
| 251 | -42.90 | -45.00 | 43.00 | DAT |
| 252 | -51.70 | -30.90 | 39.90 | DAT |
| 253 | -43.60 | 36.30 | 8.50 | DAT |
| 254 | -20.40 | -64.60 | 51.40 | DAT |
| 255 | -25.80 | -65.00 | 32.20 | DAT |
| 256 | -9.90 | -56.90 | 59.80 | DAT |
| 257 | -7.10 | -63.70 | 54.90 | DAT |
| 258 | -30.00 | -74.10 | 36.10 | DAT |
| 259 | -46.20 | -57.70 | -7.90 | DAT |
| 260 | -45.20 | 2.70 | 32.40 | DAT |
| 261 | -34.70 | 5.60 | 34.00 | DAT |
| 262 | -37.60 | 38.40 | 17.20 | DAT |
| 263 | -41.60 | 8.70 | 22.20 | DAT |
| 264 | -35.70 | 33.10 | 32.00 | DAT |
| 265 | 10.30 | -57.30 | 58.30 | DAT |
| 266 | 29.20 | 1.90 | 52.40 | DAT |
| 267 | 29.90 | -7.80 | 47.40 | DAT |
| 268 | 22.60 | 5.60 | 57.60 | DAT |
| 269 | 38.80 | -42.60 | 40.40 | DAT |
| 270 | 36.80 | 37.80 | 13.10 | DAT |
| 271 | 48.10 | 38.40 | 2.40 | DAT |
| 272 | 23.00 | -66.40 | 51.80 | DAT |
| 273 | 32.30 | -63.60 | 33.80 | DAT |
| 274 | 33.50 | -48.20 | 49.40 | DAT |
| 275 | 57.00 | -53.80 | -1.10 | DAT |
| 276 | 47.30 | 2.00 | 37.60 | DAT |
| 277 | 46.60 | 7.80 | 19.30 | DAT |
| 278 | -14.40 | -57.80 | 18.40 | RST |
| 279 | -8.80 | -49.80 | 4.20 | RST |
| 280 | -33.80 | -33.20 | -15.40 | RST |
| 281 | -22.50 | -37.10 | -15.00 | RST |
| 282 | 13.80 | -54.10 | 10.90 | RST |
| 283 | 34.60 | -35.60 | -12.30 | RST |
| 284 | 34.60 | -23.90 | -20.40 | RST |
| 285 | 24.50 | -36.20 | -13.20 | RST |

*Note*. SMN, sensorimotor networks; CON, cingulo-opercular network; CPN: Cingulo-Parietal network; AUD, auditory network; DMN, default mode network; VIN, visual network; FPN, fronto-parietal network; SAN, salience network; VAN, ventral attention network; DAN, dorsal attention network; RST: Retrosplenial temporal network.

**Supplementary file 1S.** **Graph-analysis results using 285 ROIs from the Gordon atlas.**

| **Duration of time window** | **Threshold** | ***E_g_*** | | ***E_loc_*** | | **PC** | | **Modularity** | |
| --- | --- | --- | --- | --- | --- | --- | --- | --- | --- |
|  |  | *z* | *P* | *z* | *P* | *z* | *P* | *z* | *P* |
| 41TR (30 s) | 0.10 | 4.0764 | 0.0001 | 0.9014 | 0.3674 | 2.8656 | 0.0042 | 3.1077 | 0.0019 |
|  | 0.15 | 4.1302 | 0.0001 | -0.4978 | 0.6186 | 1.8431 | 0.0653 | 2.8925 | 0.0038 |
|  | 0.20 | 4.1571 | 0.0001 | -3.1077 | 0.0019 | 1.8431 | 0.0653 | 2.7311 | 0.0063 |
|  | 0.25 | 4.1840 | 0.0001 | -4.0764 | 0.0000 | 3.2692 | 0.0011 | 2.3813 | 0.0173 |
| 50TR (36 s) | 0.10 | 3.7266 | 0.0002 | 2.2736 | 0.0230 | 1.8431 | 0.0653 | 2.9463 | 0.0032 |
|  | 0.15 | 3.7535 | 0.0002 | -0.2556 | 0.7982 | 1.4395 | 0.1500 | 2.7311 | 0.0063 |
|  | 0.20 | 3.8342 | 0.0001 | -3.4575 | 0.0005 | 1.8162 | 0.0693 | 2.5965 | 0.0094 |
|  | 0.25 | 4.0495 | 0.0001 | -3.9419 | 0.0001 | 2.0584 | 0.0396 | 2.5427 | 0.0110 |
| 62TR (45 s) | 0.10 | 4.0226 | 0.0001 | 2.8925 | 0.0038 | 2.0584 | 0.0396 | 3.6997 | 0.0002 |
|  | 0.15 | 4.0764 | 0.0001 | -0.3902 | 0.6964 | 1.9777 | 0.0480 | 3.6459 | 0.0003 |
|  | 0.20 | 4.1033 | 0.0001 | -3.0270 | 0.0025 | 1.9508 | 0.0511 | 3.2423 | 0.0012 |
|  | 0.25 | 4.1033 | 0.0001 | -3.8342 | 0.0001 | 2.6772 | 0.0074 | 2.9194 | 0.0035 |
| 83TR (60 s) | 0.10 | 3.5114 | 0.0004 | 1.4395 | 0.1500 | 2.3544 | 0.0186 | 3.3230 | 0.0009 |
|  | 0.15 | 3.6459 | 0.0003 | -1.5741 | 0.1155 | 2.0584 | 0.0396 | 3.1616 | 0.0016 |
|  | 0.20 | 3.6459 | 0.0003 | -3.3499 | 0.0008 | 2.8925 | 0.0038 | 2.8118 | 0.0049 |
|  | 0.25 | 3.5114 | 0.0004 | -3.8073 | 0.0001 | 1.9508 | 0.0511 | 2.4351 | 0.0149 |

*Notes*. Statistics are derived from Wilcoxon signed-rank tests. Graph metrics (*E_g_*: global efficiency; *E_loc_*: local efficiency; PC: participation coefficient; Modularity) were computed at the entire-network level.

**Supplementary file 1T.** **Graph-analysis results using 226 ROIs from the Power atlas combining with the bilateral hippocampus ROIs derived from Kim’s meta-analysis.**

| **Duration of time window** | **Threshold** | ***E_g_*** | | ***E_loc_*** | | **PC** | | **Modularity** | |
| --- | --- | --- | --- | --- | --- | --- | --- | --- | --- |
|  |  | *z* | *P* | *z* | *P* | *z* | *P* | *z* | *P* |
| 41TR (30 s) | 0.10 | 4.1840 | 0.0001 | 0.0673 | 0.9464 | 2.5158 | 0.0119 | 2.5965 | 0.0094 |
|  | 0.15 | 4.2917 | 0.0001 | -1.3050 | 0.1919 | 2.5696 | 0.0102 | 2.5427 | 0.0110 |
|  | 0.20 | 4.3186 | 0.0001 | -3.1347 | 0.0017 | 2.3005 | 0.0214 | 2.1660 | 0.0303 |
|  | 0.25 | 4.2647 | 0.0001 | -3.9957 | 0.0001 | 2.1122 | 0.0347 | 1.8969 | 0.0578 |
| 50TR (36 s) | 0.10 | 3.8342 | 0.0001 | 1.4395 | 0.1500 | 3.1616 | 0.0016 | 3.0539 | 0.0023 |
|  | 0.15 | 4.0226 | 0.0001 | -0.5516 | 0.5812 | 2.7041 | 0.0068 | 2.5427 | 0.0110 |
|  | 0.20 | 3.9957 | 0.0001 | -2.4351 | 0.0149 | 2.4620 | 0.0138 | 2.1122 | 0.0347 |
|  | 0.25 | 4.1571 | 0.0001 | -3.7535 | 0.0002 | 1.8431 | 0.0653 | 1.7086 | 0.0875 |
| 62TR (45 s) | 0.10 | 4.2378 | 0.0001 | 1.9508 | 0.0511 | 3.2961 | 0.0010 | 3.3230 | 0.0009 |
|  | 0.15 | 4.2647 | 0.0001 | -0.2018 | 0.8401 | 3.1347 | 0.0017 | 3.2423 | 0.0012 |
|  | 0.20 | 4.2917 | 0.0001 | -2.8925 | 0.0038 | 2.8656 | 0.0042 | 2.7311 | 0.0063 |
|  | 0.25 | 4.2647 | 0.0001 | -3.9419 | 0.0001 | 2.4620 | 0.0138 | 2.0046 | 0.0450 |
| 83TR (60 s) | 0.10 | 3.0539 | 0.0023 | 0.3363 | 0.7366 | 3.2154 | 0.0013 | 3.2154 | 0.0013 |
|  | 0.15 | 3.5652 | 0.0004 | -1.4395 | 0.1500 | 2.8925 | 0.0038 | 2.5696 | 0.0102 |
|  | 0.20 | 3.6997 | 0.0002 | -2.4082 | 0.0160 | 2.5696 | 0.0102 | 2.6772 | 0.0074 |
|  | 0.25 | 3.5921 | 0.0003 | -3.4575 | 0.0005 | 2.2467 | 0.0247 | 2.4351 | 0.0149 |

*Notes*. Statistics are derived from Wilcoxon signed-rank tests. Graph metrics (*E_g_*: global efficiency; *E_loc_*: local efficiency; PC: participation coefficient; Modularity) were computed at the entire-network level.

**Supplementary file 1U. Edge stability analysis.**

| **Threshold** | **High encoding state**  **(Real vs. Randomized)** | | **Low encoding state**  **(Real vs. Randomized)** | | **High vs. low encoding states** | |
| --- | --- | --- | --- | --- | --- | --- |
|  | ***z*** | ***P*** | ***z*** | ***P*** | ***z*** | ***P*** |
| 0.1 | 4.3724 | 0.00001 | 4.3724 | 0.00001 | 2.3006 | 0.0214 |
| 0.15 | 4.3724 | 0.00001 | 4.3724 | 0.00001 | 1.2916 | 0.1965 |
| 0.2 | 4.3724 | 0.00001 | 4.3724 | 0.00001 | 2.3006 | 0.0214 |
| 0.25 | 4.3724 | 0.00001 | 4.3724 | 0.00001 | 2.2198 | 0.0264 |

*Notes.* Statistics are derived from Wilcoxon signed-rank tests. Comparisons between the real and randomized networks gave the same results in most cases because the signed ranks were identical.

**Supplementary file 1V. Results of additional analysis using adjusted graph metrics.** We computed “adjusted” graph metrics by regressing out overall FC on a window-by-window basis.

| **Duration of time window** | **Threshold** | ***E_g_*** | | ***E_loc_*** | | **PC** | | **Modularity** | |
| --- | --- | --- | --- | --- | --- | --- | --- | --- | --- |
|  |  | *z* | *P* | *z* | *P* | *z* | *P* | *z* | *P* |
| 36 s | 0.10 | 0.4440 | 0.6571 | 1.8969 | 0.0578 | -1.2243 | 0.2209 | 2.5427 | 0.0110 |
|  | 0.15 | 0.3363 | 0.7366 | 1.4126 | 0.1578 | -1.0897 | 0.2758 | 2.0853 | 0.0370 |
|  | 0.20 | 0.0673 | 0.9464 | 1.4395 | 0.1500 | -0.8207 | 0.4118 | 2.1391 | 0.0324 |
|  | 0.25 | 0.4709 | 0.6377 | 0.4709 | 0.6377 | -0.4978 | 0.6186 | 2.0315 | 0.0422 |

*Notes*. Statistics are derived from Wilcoxon signed-rank tests. Graph metrics (*E_g_*: global efficiency; *E_loc_*: local efficiency; PC: participation coefficient; Modularity) were computed at the entire-network level.**Supplementary file 1W. Graph-analysis results from the group of 13 subjects showing the minimal difference in FD** **between high and low encoding states.**

| **Duration of time window** | **Threshold** | ***E_g_*** | | ***E_loc_*** | | **PC** | | **Modularity** | |
| --- | --- | --- | --- | --- | --- | --- | --- | --- | --- |
|  |  | **Signed rank** | ***P*** | **Signed rank** | ***P*** | **Signed rank** | ***P*** | **Signed rank** | ***P*** |
| 41TR (30 s) | 0.10 | 90 | 0.0005 | 48 | 0.8926 | 69 | 0.1099 | 60 | 0.3396 |
|  | 0.15 | 90 | 0.0005 | 36 | 0.5417 | 46 | 1.0000 | 59 | 0.3757 |
|  | 0.20 | 90 | 0.0005 | 17 | 0.0479 | 43 | 0.8926 | 63 | 0.2439 |
|  | 0.25 | 91 | 0.0002 | 3 | 0.0012 | 48 | 0.8926 | 61 | 0.3054 |
| 50TR (36 s) | 0.10 | 82 | 0.0081 | 62 | 0.2734 | 78 | 0.0215 | 71 | 0.0803 |
|  | 0.15 | 83 | 0.0061 | 36 | 0.5417 | 80 | 0.0134 | 65 | 0.1909 |
|  | 0.20 | 83 | 0.0061 | 20 | 0.0803 | 62 | 0.2734 | 61 | 0.3054 |
|  | 0.25 | 86 | 0.0024 | 9 | 0.0081 | 71 | 0.0803 | 57 | 0.4548 |
| 62TR (45 s) | 0.10 | 88 | 0.0012 | 53 | 0.6355 | 73 | 0.0574 | 71 | 0.0803 |
|  | 0.15 | 89 | 0.0007 | 32 | 0.3757 | 78 | 0.0215 | 68 | 0.1272 |
|  | 0.20 | 90 | 0.0005 | 12 | 0.0171 | 80 | 0.0134 | 63 | 0.2439 |
|  | 0.25 | 91 | 0.0002 | 3 | 0.0012 | 82 | 0.0081 | 62 | 0.2734 |
| 83TR (60 s) | 0.10 | 78 | 0.0215 | 47 | 0.9460 | 36 | 0.5417 | 76 | 0.0327 |
|  | 0.15 | 80 | 0.0134 | 39 | 0.6848 | 30 | 0.3054 | 73 | 0.0574 |
|  | 0.20 | 83 | 0.0061 | 19 | 0.0681 | 46 | 1.0000 | 72 | 0.0681 |
|  | 0.25 | 83 | 0.0061 | 11 | 0.0134 | 61 | 0.3054 | 67 | 0.1465 |

*Notes*. Statistics are derived from Wilcoxon signed-rank tests. Graph metrics (*E_g_*: global efficiency; *E_loc_*: local efficiency; PC: participation coefficient; Modularity) were computed at the entire-network level.
